# Gastroenteritis Forecasting Assessing the Use of Web and Electronic Health Record Data With a Linear and a Nonlinear Approach: Comparison Study

**DOI:** 10.2196/34982

**Published:** 2023-01-31

**Authors:** Canelle Poirier, Guillaume Bouzillé, Valérie Bertaud, Marc Cuggia, Mauricio Santillana, Audrey Lavenu

**Affiliations:** 1 Department of Pediatrics Harvard Medical School Boston, MA United States; 2 Computational Health Informatics Program Boston Children's Hospital Boston, MA United States; 3 Institut national de la santé et de la recherche médicale U1099 Rennes France; 4 Laboratoire Traitement du Signal et de l'Image Université de Rennes 1 Rennes France; 5 Centre de Données Cliniques Centre Hospitalier Universitaire Rennes Rennes France; 6 Harvard Tseng-Hsi Chan School of Public Health Boston, MA United States; 7 Machine Intelligence Group for the Betterment of Health and the Environment Network Science Institute Northeastern University Boston, MA United States; 8 Faculté de médecine Université de Rennes 1 Rennes France; 9 Institut de Recherche Mathématique de Rennes Rennes France; 10 Institut national de la santé et de la recherche médicale CIC 1414 Université de Rennes 1 Rennes France

**Keywords:** infectious disease, acute gastroenteritis, modeling, modeling disease outbreaks, machine learning, public health, machine learning in public health, forecasting, digital data

## Abstract

**Background:**

Disease surveillance systems capable of producing accurate real-time and short-term forecasts can help public health officials design timely public health interventions to mitigate the effects of disease outbreaks in affected populations. In France, existing clinic-based disease surveillance systems produce gastroenteritis activity information that lags real time by 1 to 3 weeks. This temporal data gap prevents public health officials from having a timely epidemiological characterization of this disease at any point in time and thus leads to the design of interventions that do not take into consideration the most recent changes in dynamics.

**Objective:**

The goal of this study was to evaluate the feasibility of using internet search query trends and electronic health records to predict acute gastroenteritis (AG) incidence rates in near real time, at the national and regional scales, and for long-term forecasts (up to 10 weeks).

**Methods:**

We present 2 different approaches (linear and nonlinear) that produce real-time estimates, short-term forecasts, and long-term forecasts of AG activity at 2 different spatial scales in France (national and regional). Both approaches leverage disparate data sources that include disease-related internet search activity, electronic health record data, and historical disease activity.

**Results:**

Our results suggest that all data sources contribute to improving gastroenteritis surveillance for long-term forecasts with the prominent predictive power of historical data owing to the strong seasonal dynamics of this disease.

**Conclusions:**

The methods we developed could help reduce the impact of the AG peak by making it possible to anticipate increased activity by up to 10 weeks.

## Introduction

### Background

Acute gastroenteritis (AG) is a major public health problem worldwide [1]. Commonly defined as diarrhea or vomiting in the past 24 hours [2], AG is one of the main causes of morbidity and mortality among young people and causes up to 2.5 million deaths per year in children aged <5 years around the world [3]. Although it is generally a mild disease, its morbidity and economic burden are high [4]. In France, there are >21 million episodes of AG each year [5]. Although AG episodes occur throughout the year, there is a winter peak, mainly owing to norovirus and rotavirus [6,7]. During these peaks, the increase of visits to general practitioners and emergency or pediatric departments causes health care system disruptions [8].

Disease surveillance systems capable of producing accurate real-time and short-term forecasts can help public health officials design timely public health interventions to mitigate the effects of disease outbreaks in affected populations. In France, all acute diarrhea cases seen during medical appointments are reported weekly by volunteer outpatient health care providers. An estimation of AG incidence rate is then computed, at the national or regional scale, by considering the number of sentinel physicians and the medical density of the area of interest [9]. However, data collection, processing, aggregation, and distribution processes introduce up to 3 weeks of delay in the availability of AG activity information. This temporal data gap prevents public health officials from having a timely perspective about AG activity and thus leads to the design of interventions that do not take into consideration the most recent changes in disease dynamics. Therefore, there is a growing interest in finding new ways to mitigate this information gap [10,11].

To alleviate this time lag, several studies have proposed approaches to produce accurate and reliable real-time disease activity estimates, for example, to monitor influenza [11-14]. For AG, studies have been focused on identifying the clinical characteristics of the disease. Norovirus and rotavirus are the viruses responsible for most gastroenteritis outbreaks [6,7,15-18]. This disease has a strong wintertime seasonality, but this seasonality could be affected by the climate change, which would affect norovirus transmission, host’s susceptibility to norovirus infection, and resistance of norovirus to environmental conditions. This may cause large oscillations in the number of cases per year [6,7]. AG remains as a major cause of hospitalizations, especially for children, and the use of a vaccine could help to decrease the impact of the disease [16,18]. Some research teams have assessed the correlation between data sources (eg, drug reimbursement data and emergency department visits) and general practitioner visits for AG [3,19]. Other studies have shown a significant correlation between internet search query trends and AG incidence rates in different locations such as the United States, Mexico, the United Kingdom, and France [20,21]. However, none, to the best of our knowledge [22], have proposed a feasible methodology to forecast AG activity. Through this study, we investigated the challenges of achieving this and proposed a reliable forecasting approach.

### State of the Art

Existing forecasting systems for other disease outbreaks, such as influenza, include statistical models that leverage information available in near real time [11-14]. One of the first and prominent studies is Google Flu Trends [23], a web-based service operated by Google. Created in 2009, the platform used the volume of selected Google search terms to estimate influenza activity in real time. However, the web service was stopped following several prediction errors owing to changes in people’s search behavior as a result of the exceptional nature of the pandemic or owing to the announcement of a pandemic that finally did not appear [24]. Following this, some authors updated the Google Flu Trends algorithm to improve influenza forecasting, by including data from Google Correlate and Google Trends web services and other sources, for instance, historical influenza information [11]. Internet is not the only data source that can be used to produce information in real time. With the widespread adoption of patient electronic health records (EHRs), hospitals also generate a huge amount of data. Bouzillé et al [25] showed that EHRs are strongly correlated with influenza incidence rates. Some authors proposed statistical models using EHRs to predict influenza incidence rates in real time [12,26]. In addition, other studies showed that internet users’ searches were strongly correlated with influenza epidemics and other diseases, including AG [8,21].

In this study, we evaluated the feasibility of using internet search query trends and EHR to predict AG incidence rates in near real time, at the national and regional scales, and for long-term forecasts (up to 10 weeks). We used 2 different methods—a linear approach using Elastic Net and a nonlinear approach using random forest (RF). In addition, as AG outbreaks cause disruptions in hospitals and emergency departments, we estimated AG incidence rates at the level of emergency departments and hospital stays.

## Methods

### Variables to Be Predicted

#### National Level

We obtained the national (Metropolitan France) acute diarrhea weekly incidence rates (per 100,000 inhabitants) from the French Sentinel network [27], from January 2008 to March 2018. We retrieved these data in April 2018.

#### Regional Level

We obtained the regional (Brittany region) acute diarrhea incidence rates (per 100,000 inhabitants) from the French Sentinel network [27], from January 2008 to March 2018. We chose the Brittany region as we used her data from a hospital in Brittany. We retrieved these data in April 2018.

### Predictive Variables

#### Web Data

We obtained the frequency per week of the 100 most correlated French queries from Google Correlate [28]. For each signal to be predicted (national and regional levels), we retrieved Google Correlate data for the period from January 2008 to March 2018. As our prediction period is from May 2014 to February 2018, the correlation was calculated from January 2008 to April 2014. All signals were normalized to obtain mean 0 and SD 1 before calculating the correlation. The reason to correlate was to choose the most appropriate queries to predict the outbreak without previous knowledge [29]. The most correlated queries obtained for national and regional levels can differ because the weekly incidence rates for France and Brittany are different.

#### Clinical Data

We used data from the clinical data warehouse (CDW) of Rennes University Hospital (France), called entrepôt de données de l’HÔPital (eHOP). This CDW includes structured (laboratory test results, prescriptions, and International Statistical Classification of Diseases and Related Health Problems 10th Revision diagnoses) and unstructured (discharge letter, pathology reports, and operative reports) patients’ data from 1.2 million inpatients and outpatients and 45 million documents. To identify patients with specific criteria, eHOP has its own search engine system that allows to query unstructured data with keywords or structured data with codes based on terminologies.

First, to retrieve clinical data connected with AG, we performed different full-text queries (related to gastroenteritis, its symptoms, virus, or treatments). These queries allowed to obtain all documents matching with the search criteria (often, several documents for 1 patient and 1 stay). Then, for each week, we kept the oldest document for 1 patient and 1 hospital stay, and we calculated the number of hospital stays with at least one document mentioning the keyword contained in the query. As we used 19 keywords, we obtained 19 variables from CDW eHOP.

Then, we built a database containing the time series constructed from the structured data (total n=1,335,347 time series). Regrading Google Correlate, we calculated the Pearson correlation between both national and regional incidence rates and the time series from the database. We retrieved the 100 most correlated signals. As our prediction period is from May 2014 to February 2018, we calculated the correlation between January 2008 and April 2014.

Overall, we obtained 119 variables (n=19, 15.9% of variables from the full-text queries and n=100, 84% of the most correlated variables from the structured data). The 100 most correlated variables can be different for national and regional levels. We retrieved EHR data for the period from January 2008 to March 2018 in April 2018. All these data could be extracted in real time if needed.

#### Historical Data

We used the incidence rates for the previous 52 weeks as predictive variables, for both national and regional levels.

### Ethics Approval

This study was approved by the local ethics committee of the Rennes Academic Hospital (approval number 16.69).

### Statistical Models

#### Linear Approach

To minimize the negative effects of using a large number of input variables, potentially including redundant information, we used Elastic Net, a regularized multivariate regression methodology that can identify parsimonious models [30]. Elastic Net combines the power of Lasso and Ridge regressions, allowing to perform a variable selection on variables that are highly correlated [31,32]. We performed the Elastic Net regression analysis using the *caret* package in R (R Foundation for Statistical Computing) and the associated function fit with the *glmnet* method [33,34]. We fixed a coefficient λ=0.5 to give the same importance to Ridge and Lasso methods.

The formulation of our model is the following:



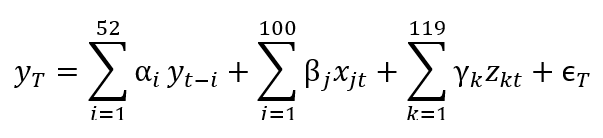



Here, yT denotes AG incidence rate at time T=t, t+1, t+2, t+3 (for the different levels of prediction), 
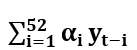
 denotes historical variables, 
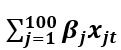
 denotes Google data, 
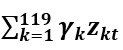
 denotes EHR data, and 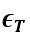 denotes residuals.

For a given week, we needed to find the parameters, α=(α_1_,..α_52_), β=(β_1_,..β_100_), and γ=(γ_1_,..γ_119_), that minimize the following:



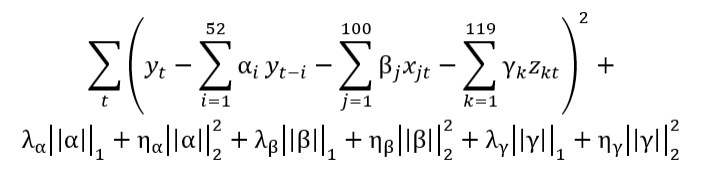



Here, 
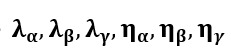
 are hyperparameters of the Elastic Net regression. We used 10-block cross-validation to optimize the parameters. All parameters (α=[α_1_,..α_52_], β=[β_1_,..β_100_], and γ=[γ_1_,..γ_119_]) were dynamically trained every week with a rolling window using all data available. In this way, the size of our training data set increased every week. For example, for the first week of January 2015, our training data set ranged from January 2008 to the last week of December 2014. To predict the first week of January 2016, our training data set ranged from January 2008 to the last week of December 2015. We obtained estimates from May 2014 to February 2018.

#### Nonlinear Approach

RF is a nonlinear machine learning approach based on the construction of multiple decision trees using the general bootstrap aggregating technique (known as bagging) [35]. We used this method as it showed good performance in short-term forecasting even when it is compared with other machine learning approaches such as support vector machine or neural network or a traditional approach such as autoregressive integrated moving average [36,37].

With RF, the AG incidence rates are obtained with the following:
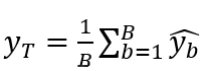


Here, y_T_ denotes AG incidence rate at time T=t, t+1, t+2, t+3 (for the different levels of prediction) and 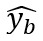 denotes AG incidence rates estimate obtained with the decision tree b. We used the R package, *randomForest* [38], to create our RF models. The hyperparameters corresponding to the number of decision trees and the number of variables randomly sampled at each split were optimized on a training data set from January 2008 to May 2014. Then, regarding the Elastic Net model, RF was dynamically recalibrated for every new week of prediction by incorporating all the data available. We obtained estimates from May 2014 to February 2018.

#### Contribution of Each Data Source

In addition, to assess the contribution of each individual data sources or their combinations, we built Elastic Net and RF models using the following predictive variables:

1. AG incidence rates—baseline model called autoregressive model of order 52 (AR(52)) in the following sections—for the previous 52 weeks
2. Google data
3. EHR data
4. Google data and AR(52)
5. EHR data and AR(52)
6. Google data and EHR data

### Evaluation

To assess the performance of our models, we compared our estimates with the real incidence rates from the Sentinel network. We calculated the root mean squared error and the Pearson correlation coefficient for our test period starting from May 2014 to February 2018. The model allowing to obtain the most accurate estimates is the one having the highest correlation and the lowest error:

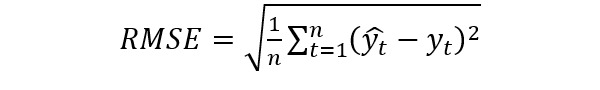

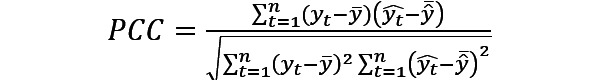

Here, 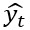 is the predicted value for the week t, 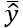 is the mean of predicted values, y_t_ is the real value for the week t, and 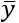 is the mean of real values.

### Comparison With Influenza

As we used a method developed for influenza outbreaks, we compared the results obtained for AG with those obtained for influenza. The aim was to determine whether external data sources are as relevant for AG as for influenza. We started by comparing the stationarity and the seasonality of both time series by calculating the following:

1. The autocorrelation function (ACF), allowing to determine the autocorrelation between y_t_ and y_t–h_:



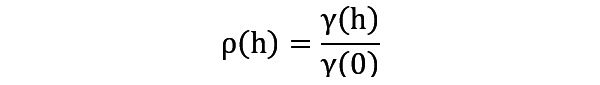



where γ(h)=cov(y_t_, y_t–h_)

2. The partial ACF (PACF), allowing to determine the autocorrelation between y_t_ and y_t–h_ after removing the autocorrelation between the intermediate variables y_t–1_,...,y_t–h+1_:

r(h)=corr(y_t_,y_t–h_|y_t–1_,...,y_t–h+1_)

Then, we compared the accuracy of estimates for forecast up to 10 weeks with Elastic Net and RF models using only historical data or combining Google, EHR, and historical data.

## Results

### Overview

First, we studied the impact of each data source for short-term forecasts with the 2 different approaches already used to predict influenza outbreaks—a linear approach with the Elastic Net model and a nonlinear approach with an RF model.

Then, we analyzed the AG and influenza time series, especially the seasonality, to better understand the differences between the 2 diseases.

Finally, we compared AG and influenza results obtained for long-term forecasts with the 2 approaches, and we assessed the impact of external data sources to increase the accuracy of our estimates.

### Linear Approach

#### Overview

At the national and regional levels, in terms of error, the lowest values are obtained with models using historical data and external data sources (Table 1). At the national level, in terms of error, both data sources, Google and EHR produce the most accurate estimates compared with the model using only historical data—AR (52). At the regional level, the model using only historical data and EHR allows to obtain lower errors than the model using historical data and both Google and EHR data.

In terms of correlation, in most cases, at the national and regional levels, the model using only historical data allows to obtain the highest values.

**Table 1:** PCC^a^ and RMSE^b^ values obtained for the entire prediction period (May 2014 to March 2018) at the national and regional levels, with all the combinations of data sources.

Levels and data sources		Real time		1-week forecast		2-week forecast		3-week forecast	
		PCC	RMSE	PCC	RMSE	PCC	RMSE	PCC	RMSE
**National**									
	AR(52)^c^	*0.946* ^d^	*16.16*	*0.910*	22.69	*0.898*	26.95	*0.884*	30.69
	Google	0.830	42.75	0.803	44.99	0.801	41.27	0.770	38.96
	EHR^e^	0.477	48.35	0.512	45.59	0.489	47.37	0.519	44.65
	AR(52) and Google	*0.941*	18.10	0.896	24.17	0.871	26.98	0.847	28.24
	AR(52) and EHR	0.932	*16.41*	0.880	21.58	0.820	26.15	0.823	*25.93*
	Google and EHR	0.836	36.09	0.846	34.48	0.779	34.23	0.795	32.32
	AR(52), Google, and EHR	0.936	21.26	0.903	*20.94*	0.856	*24.16*	0.845	*25.33*
**Regional**									
	AR(52)	0.725	*40.75*	*0.705*	44.18	*0.670*	47.65	*0.681*	49.12
	Google	0.652	65.84	0.603	64.79	0.594	60.33	0.596	61.67
	EHR	0.462	59.83	0.538	55.62	0.546	55.87	0.582	52.90
	AR(52) and Google	*0.738*	42.07	0.665	46.44	0.616	47.82	0.619	47.74
	AR(52) and EHR	0.697	*40.99*	0.685	*42.38*	0.637	*46.48*	0.634	*46.31*
	Google and EHR	0.608	60.70	0.610	60.97	0.615	57.50	0.628	59.72
	AR(52), Google, and EHR	0.724	42.12	0.689	45.24	0.646	47.37	0.620	52.19

^a^PCC: Pearson correlation coefficient.

^b^RMSE: root mean squared error.

^c^AR(52): autoregressive model of order 52.

^d^Italicization highlights the 2 highest correlations and lowest errors obtained with the models for real time and 1-week, 2-week, and 3-week forecasts.

^e^EHR: electronic health record.

#### National Analysis

For real-time estimates, the error values range from 48.4 to 16.2 and the correlation values range from 0.83 to 0.95, with the lowest error and the highest correlation obtained with the model using only historical data—AR(52). For 1-week estimates, the error values range from 45.6 to 20, with the lowest error and the highest correlation obtained with the model using historical data and both external data sources, Google and EHR. In terms of correlation, the correlation values range from 0.51 to 0.91, with the highest value obtained with the model using only historical data. For 2-week and 3-week estimates, we have similar results, with error values ranging from 47.4 to 24.2 and 44.6 to 25.3, respectively, obtained with the model using historical data and both external data sources, Google and EHR. In terms of correlation, the values range from 0.49 to 0.90 and from 0.52 to 0.88, respectively, with the highest correlation obtained with AR(52) model.

**Figure 1:**
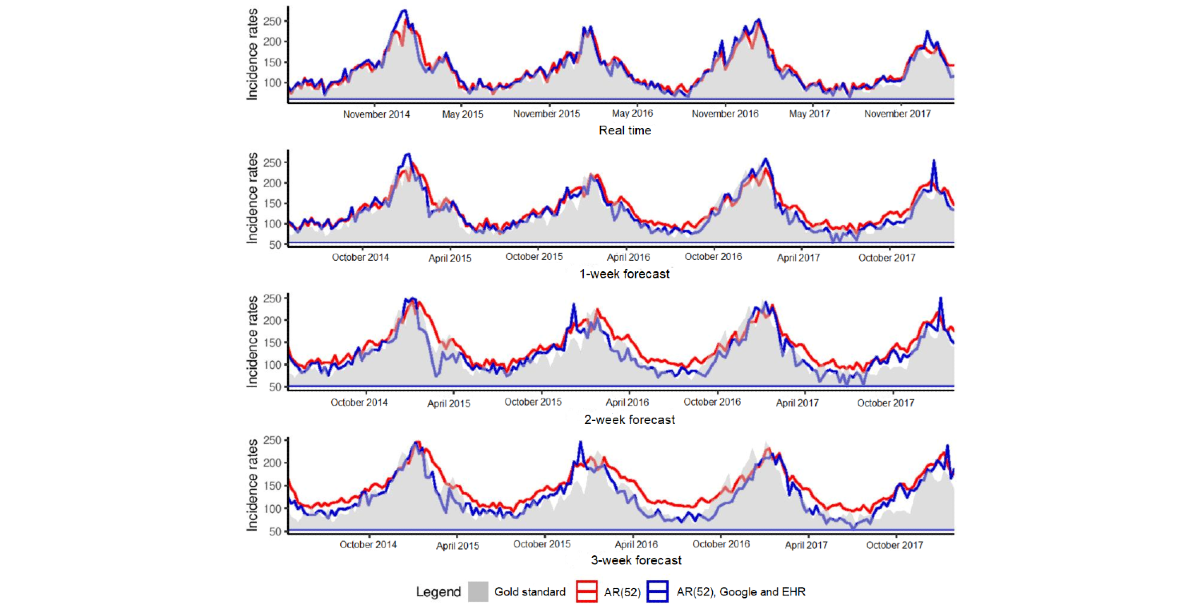
National level. Predictions up to 3 weeks obtained at the national level with the model using only historical data and the model using historical data and both data sources, Google and EHR. Gold standard, French Sentinel network data. EHR: electronic health record.

Figure 1 illustrates the estimates obtained at the national level for forecasts up to 3 weeks with the model using only historical data and the model using historical data and both data sources, Google and EHR. For real-time estimates, the results obtained with the 2 models are comparable, but for long-term forecasts (1, 2, and 3 weeks), the estimates obtained with the AR(52) model are delayed. In addition, the model using only historical data tends to smooth estimates and overestimate between peaks.

**Figure 2:**
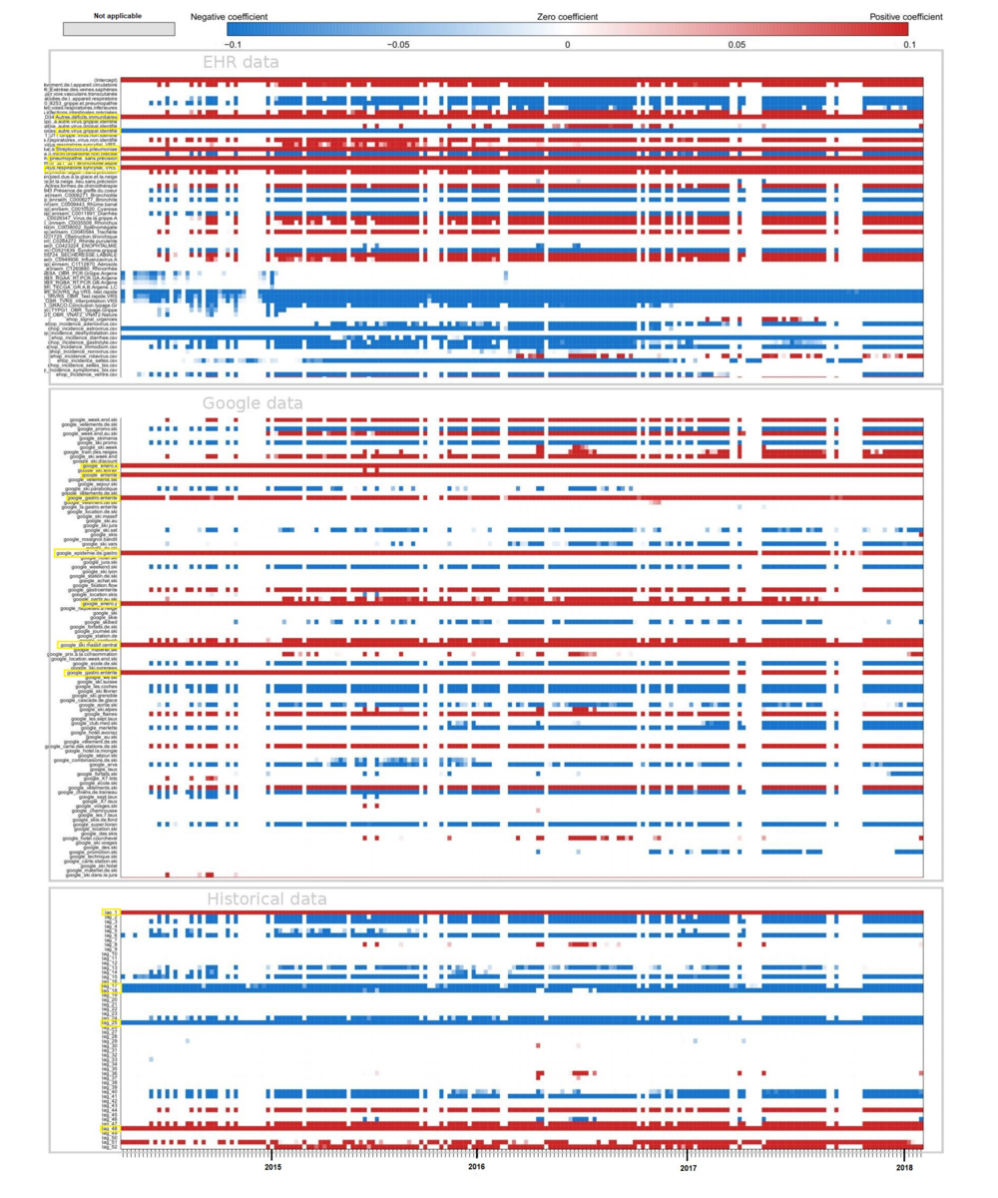
National level. Heatmap of the coefficients. Each line of the heatmap corresponds to one predictive variable used in the model and each point of the line corresponds to 1 week predicted. The first block of variables corresponds to electronic health record (EHR) data, the second one corresponds to Google data, and the third one to historical data. In blue, a negative coefficient is associated with the variable, whereas in red, it is a positive coefficient. The white color means that the predictive variable is not selected by the model and does not participate in forecasting the corresponding week. In yellow, highlighted variables that are kept by the model almost all the time. For EHR data, it corresponds to the predictive variables for the keywords “Autres deficits immunitaires,” “Autre virus grippal identifié,” “Streptococcus pneumoniae,” “Pneumopathie,” “Virus respiratoire syncytial.” For Google data, it is the keywords: “enero,” “enterite,” “epidemie de gastro,” “gastro entérite,” “ski massif central.” For historical data, it corresponds to the previous week as well as week 17, week 18, week 25, and week 48 before the one we want to predict.

Figure 2 is a visualization of the values of the coefficients for the model using historical data and both data sources, Google and EHR. For real-time estimates, the heat map shows that the model uses multiple variables from all data sources, such as historical data, Google data, and EHR data. Similar plots are presented in Multimedia Appendix 1 for long-term estimates.

#### Regional Analysis

For real-time estimates, the error values range from 65.8 to 40.8 and the correlation values range from 0.46 to 0.74, with the lowest value for the error obtained with the model using only historical data and the highest value for the correlation obtained with the model using historical data and Google data. For 1-week, 2-week, and 3-week estimates, the error values range from 64.8 to 42.4, from 60.3 to 46.5, and from 61.7 to 46.3, respectively. The lowest errors values for long-term forecasts are all obtained with the model using historical data and EHR data. In terms of 1-week, 2-week, and 3-week correlation, the values range from 0.54 to 0.71, from 0.55 to 0.67, and from 0.58 to 0.68, respectively. The highest correlations for long-term forecasts are all obtained with the model using only historical data—AR(52).

**Figure 3:**
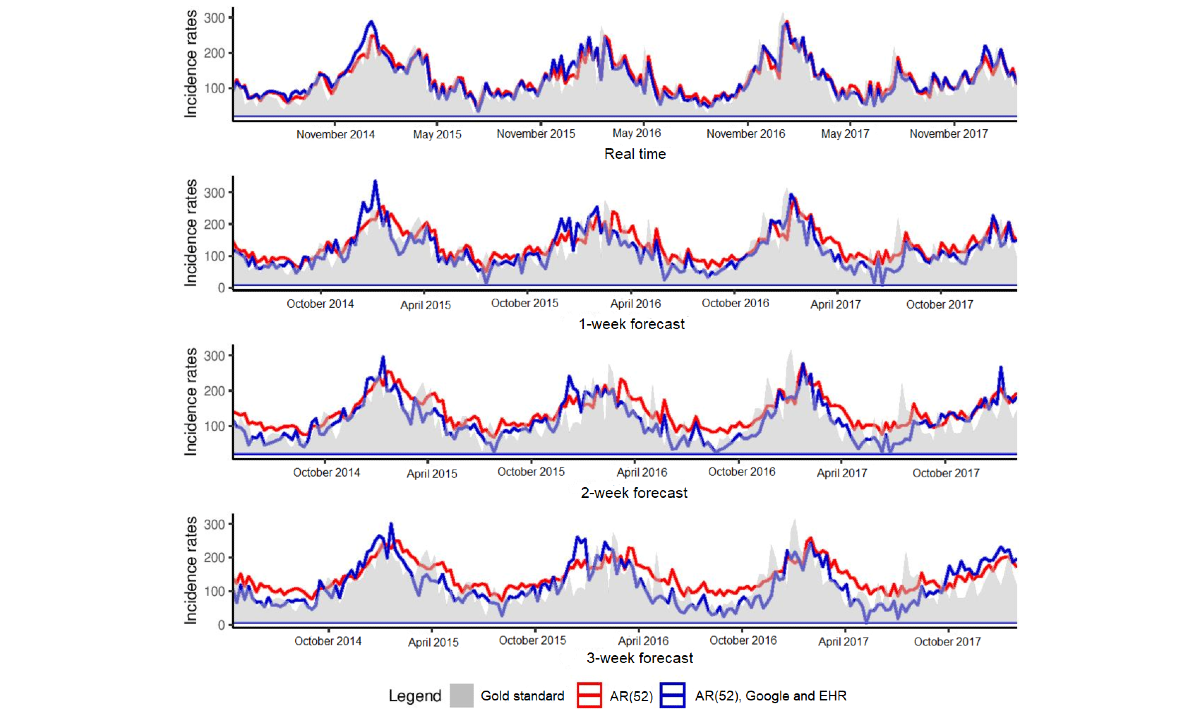
Regional level. Predictions up to 3 weeks obtained at the regional level with the model using only historical data and the model using historical data and both data sources, Google and EHR. Gold standard, French Sentinel network data. EHR: electronic health record.

Figure 3 illustrates the estimates obtained at the regional level for forecasts up to 3 weeks with the model using only historical data and the model using historical data and both data sources, Google and EHR. At the national level, for real-time estimates, the results obtained with the 2 models are comparable, but for long-term forecasts, the estimates obtained with the AR(52) model are delayed and tend to be smoothed and overestimated between peaks.

**Figure 4:**
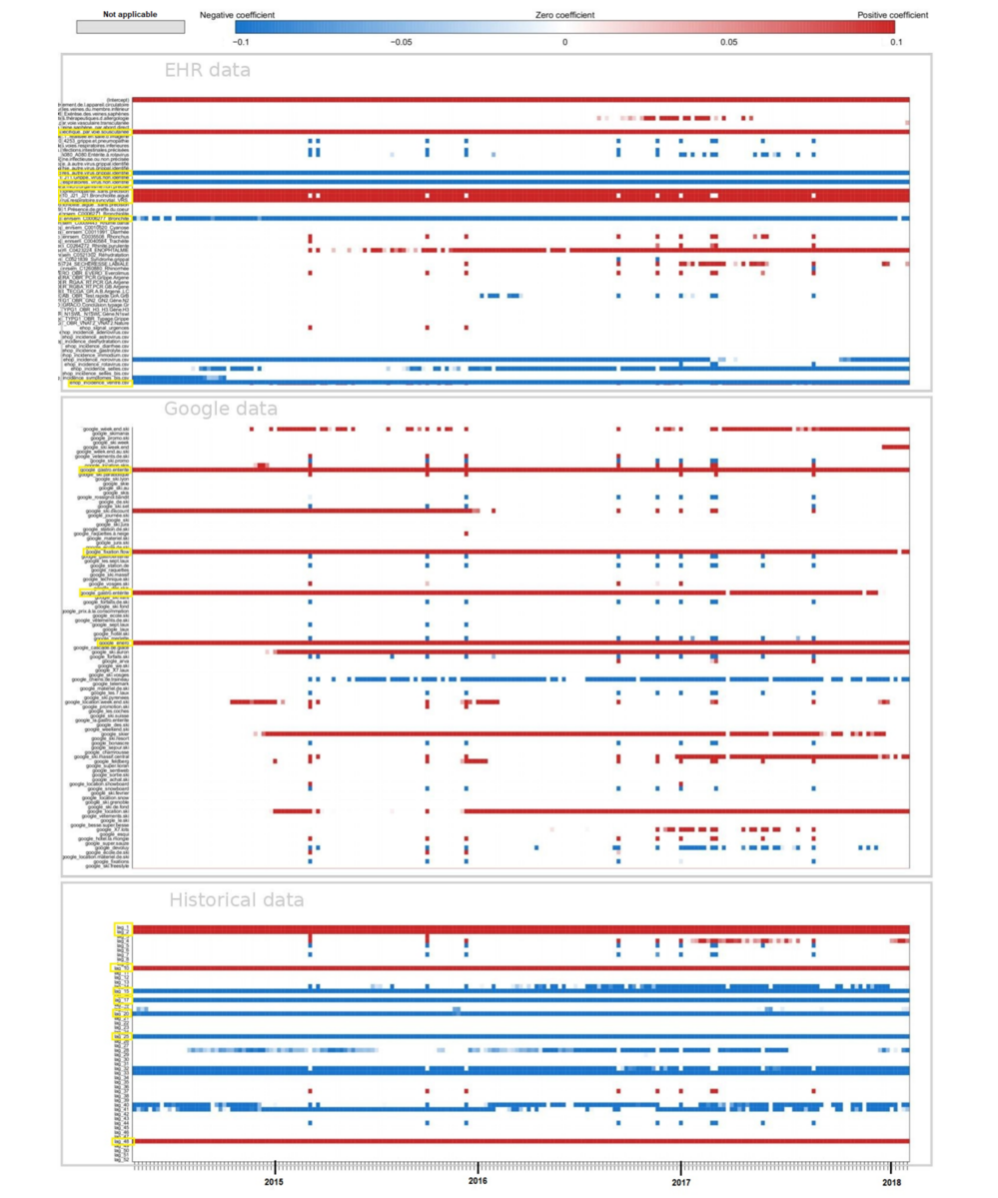
Regional level. Heatmap of the coefficients. Each line of the heatmap corresponds to one predictive variable used in the model and each point of the line corresponds to 1 week predicted. The first block of variables corresponds to electronic health record (EHR) data, the second one corresponds to Google data, and the third one to historical data. In blue, a negative coefficient is associated with the variable, whereas in red, it is a positive coefficient. The white color means that the predictive variable is not selected by the model and does not participate in forecasting the corresponding week. In yellow, highlighted variables that are kept by the model almost all the time. For EHR data it corresponds to the predictive variables for the keywords “Par voie sous cutannée,” “Autre virus grippal identifié,” “Voies respiratoires. Virus non identifié,” “Pneumopathie,” “Bronchiolite aigüe,” “Virus respiratoire syncytial,” “Bronchite,” “Ventre.” For Google data, it is the keywords: “enero,” “gastro enterite,” “gastro entérite,” “fixations.” For historical data, it corresponds to the two previous weeks as well as week 10, week 15, week 17, week 20, week 25, and week 48 before the one we want to predict.

The heat map (Figure 4) shows that for real-time estimates at the regional level, the model uses multiple variables from historical data (approximately 11 variables) and low number of variables from Google data (approximately 10 variables) and EHR data (approximately 9 variables) compared with those at the national level. Similar plots are presented in Multimedia Appendix 1 for long-term estimates.

### Nonlinear Approach

#### Overview

For the nonlinear approach, at the national level, in terms of error and correlation, results are comparable between the model using only historical data—AR(52)—and the models combining historical data and external data sources (Table 2). At the regional level, in terms of error, the lowest errors are mostly obtained with the model including historical and EHR data. In terms of correlation, the highest values are mostly obtained with the model combining historical data and both data sources, Google and EHR. For the nonlinear approach, the values for correlation are higher and the values for errors are lower than the values obtained with the linear approach.

**Table 2:** PCC^a^ and RMSE^b^ values obtained for the entire prediction period (May 2014 to March 2018) for all levels and models.

Levels and data sources		Real time		1-week forecast			2-week forecast			3-week forecast	
		PCC	RMSE	PCC	RMSE	PCC		RMSE	PCC		RMSE
**National**											
	AR(52)^c^	*0.942* ^d^	*15.47*	*0.913*	*19.71*	*0.892*		*22.19*	*0.903*		*22.30*
	Google	0.884	45.59	0.876	45.72	0.858		42.63	0.830		40.52
	EHR^e^	0.795	32.93	0.615	50.68	0.739		37.84	0.692		41.30
	AR(52) and Google	*0.946*	*15.87*	*0.913*	21.68	*0.892*		23.63	*0.909*		*22.98*
	AR(52) and EHR	0.938	*15.93*	0.906	*20.21*	0.887		*22.85*	0.890		23.31
	Google and EHR	0.833	43.26	0.780	49.50	0.849		37.70	0.790		41.88
	AR(52), Google, and EHR	*0.946*	*15.72*	0.909	21.76	*0.895*		23.87	0.886		24.11
**Regional**											
	AR(52)	0.745	*38.47*	0.699	42.68	0.685		*44.11*	0.677		45.05
	Google	0.708	62.90	0.658	61.58	0.671		57.02	0.689		54.55
	EHR	0.651	47.76	0.531	66.99	0.562		60.51	0.526		63.26
	AR(52) and Google	0.757	39.71	0.700	46.91	0.694		47.38	*0.703*		47.87
	AR(52) and EHR	0.743	*38.37*	*0.720*	*41.05*	0.694		*43.83*	0.694		*44.09*
	Google and EHR	0.542	76.87	0.584	69.17	0.663		55.48	0.658		56.25
	AR(52), Google, and EHR	*0.759*	*38.88*	*0.718*	44.63	*0.702*		46.25	*0.701*		47.17

^a^PCC: Pearson correlation coefficient.

^b^RMSE: root mean squared error.

^c^AR(52): autoregressive model of order 52.

^d^Italicization highlights the 2 highest correlations and lowest errors obtained with the models for real time and 1-week, 2-week, and 3-week forecasts.

^e^EHR: electronic health record.

#### National Analysis

For real-time estimates, the error values range from 45.6 to 15.5 and the correlation values range from 0.80 to 0.95, with the lowest error and the highest correlation obtained with the model using only historical data—AR(52)—or the models combining historical data and external data sources. The results are similar for long-term forecasts, with error values ranging from 50.7 to 19.7 and correlation values ranging from 0.62 to 0.91 for 1-week estimates. For 2-week and 3-week estimates, the error values range from 42.6 to 22.8 and 41.9 to 22.3, respectively. In terms of 2-week and 3-week correlation, the values range from 0.74 to 0.90 and from 0.69 to 0.91, respectively.

**Figure 5:**
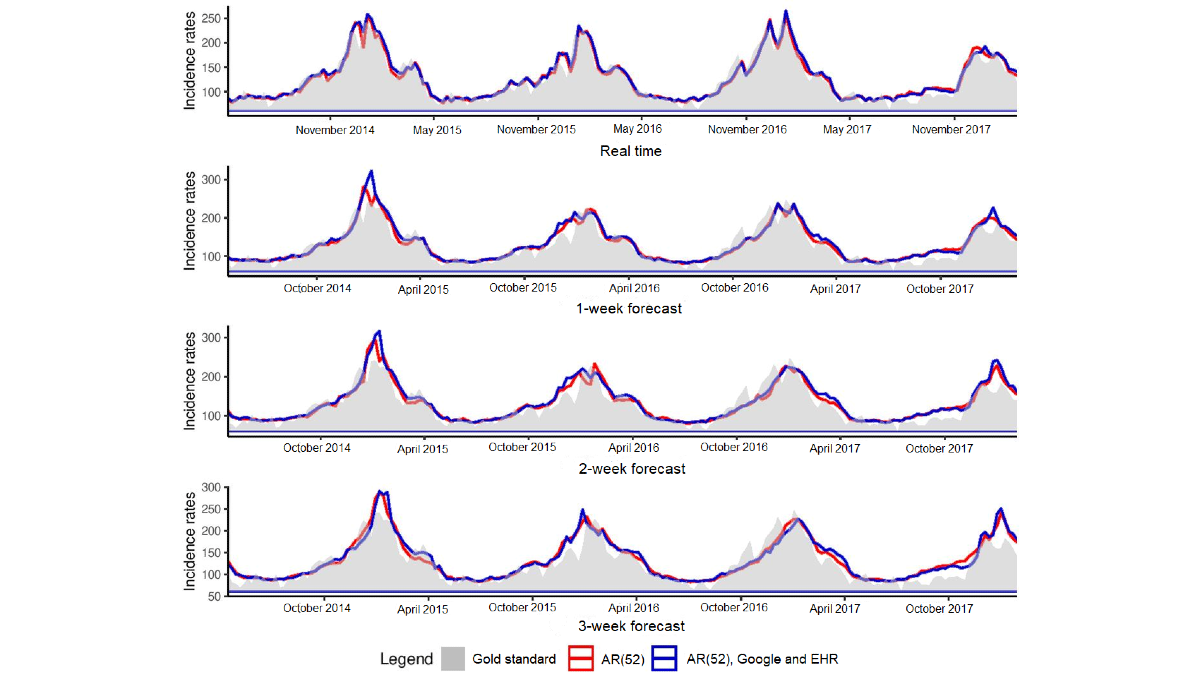
National level. Predictions up to 3 weeks obtained at the national level with the model using only historical data and the model using historical data and both data sources, Google and EHR. Gold standard, French Sentinel network data. EHR: electronic health record.

Figure 5 illustrates the estimates obtained at the national level for forecasts up to 3 weeks with the model using only historical data and the model using historical data and both data sources, Google and EHR. For real-time estimates and long-term forecasts, the results obtained with the 2 models are comparable. In comparison with the linear approach, the nonlinear approach tends to smooth estimates.

#### Regional Analysis

For real-time estimates, the error values range from 76.9 to 38.4 and the correlation values range from 0.54 to 0.76, with the lowest error and the highest correlation values obtained with AR(52) model and the models combining historical data and external data sources. For 1-week, 2-week, and 3-week estimates, the error values range from 69.2 to 41.1, from 60.5 to 43.8, and from 63.3 to 44.1, respectively. The lowest errors values for long-term forecasts are all obtained with the model using historical and EHR data. In terms of 1-week, 2-week, and 3-week correlation, the values range from 0.53 to 0.72, from 0.56 to 0.70, and from 0.53 to 0.70, respectively. The highest correlations for long-term forecasts are all obtained with the model using historical data and both data sources, Google and EHR.

**Figure 6:**
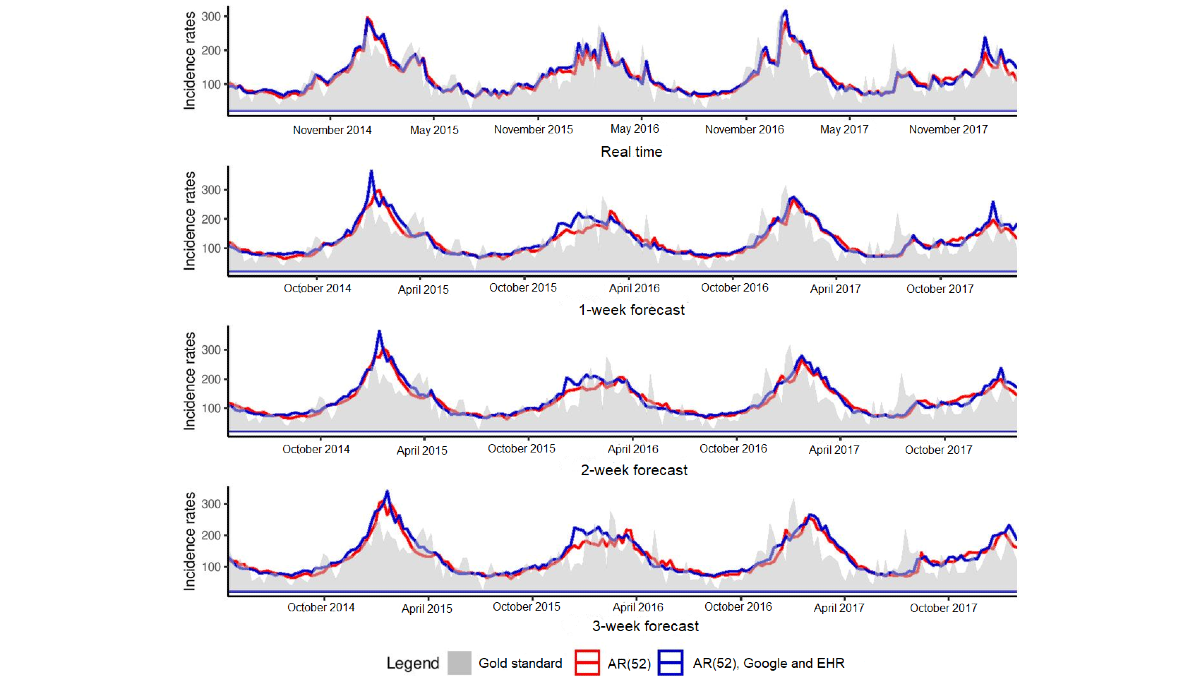
Regional level. Predictions up to 3 weeks obtained at the regional level with the model using only historical data and the model using historical data and both data sources, Google and EHR. Gold standard, French Sentinel network data. EHR: electronic health record.

Figure 6 illustrates the estimates obtained at the regional level for forecasts up to 3 weeks with the model using only historical data and the model using historical data and both data sources, Google and EHR. At the national level, results are comparable between the 2 models, and the nonlinear approach tends to smooth the estimates.

### Comparison of AG and Influenza

**Figure 7:**
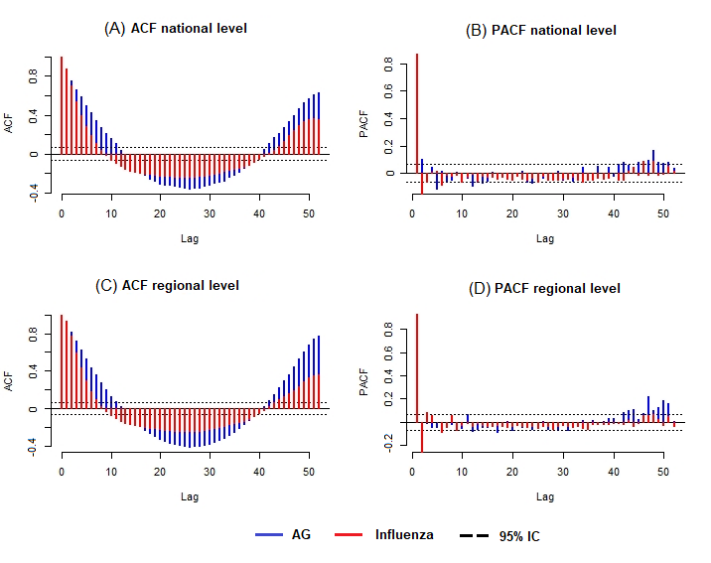
ACF and PACF. Autocorrelation obtained for flu and AG at the national level (Figures A and B) and regional level (Figures C and D). ACF: autocorrelation function; AG: acute gastroenteritis; PACF: partial autocorrelation function.

To assess the role of external data sources in AG forecasting in comparison with influenza forecasting, we studied both time series, at the national and regional levels. As both series were stationary, we compared the seasonality. Figure 7 corresponds to ACF and PACF obtained for AG and influenza.

The ACF plot provides the correlation coefficients between a time series and its lagged values. The PACF plot provides the correlation coefficients between a time series and its lagged values after removing the effects that are already explained by the previous lags.

The ACF plots at the national and regional levels (Figures 7A and 7C) show that both time series, AG and influenza, are seasonal, but with autocorrelation more important for AG than for influenza. This result can explain why historical data are able to provide more information for AG than for influenza. We have similar results for PACF plots (Figures 7B and 7D), at the national and regional levels, where the coefficients of partial autocorrelation are larger for AG than for influenza.

### Analysis of Forecast up to 10 Weeks

#### Linear Approach

**Figure 8:**
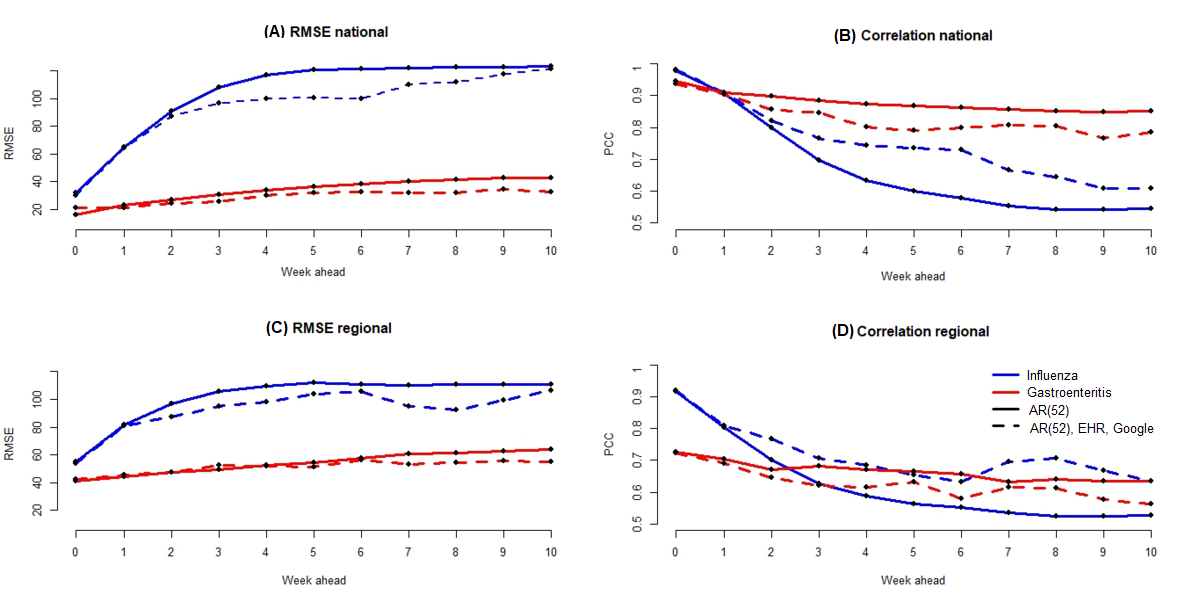
(A) Error values obtained at the national level for the flu and gastroenteritis for forecasts up to 10 weeks with the Elastic Net model. The solid line corresponds to the results obtained with the Elastic Net model using only historical data. The dotted line corresponds to the results obtained with the Elastic Net model using historical data and both Google and EHR data. The red color is the results for gastroenteritis disease, whereas the blue color is the results for the flu. This style line and color code are used for the 4 panels of this figure. (B) Correlation values obtained at the national level. (C) Error values obtained at the regional level. (D) Correlation values obtained at the regional level. EHR: electronic health record; RMSE: root mean squared error.

Figure 8 and Table S1 in Multimedia Appendix 1 show, for the linear approach, errors and correlation for AG at the national and regional levels, for forecasts up to 10 weeks. At the national level, the lowest error for real-time estimates is obtained with the linear approach using only historical data—AR(52). For long-term forecasts, from up to 1 week to up to 10 weeks, the lowest errors are obtained by using historical data and both data sources, Google and EHR. In terms of correlation, in all cases, the highest values are obtained by using only historical data. At the regional level, in terms of errors, both data sources, Google and EHR, allow to improve accuracy for forecasts from up to 4 weeks to up to 10 weeks. In terms of correlation, results are similar to those at the national level, with high values obtained by using only historical data.

Figure 8 and Table S2 in Multimedia Appendix 1 show, for the linear approach, errors and correlation for influenza at the national and regional levels, for forecasts up to 10 weeks. In contrast to AG at the national and regional levels, in terms of errors and correlation, the most accurate results are obtained by using historical data, Google data, and EHR data.

#### Nonlinear Approach

**Figure 9:**
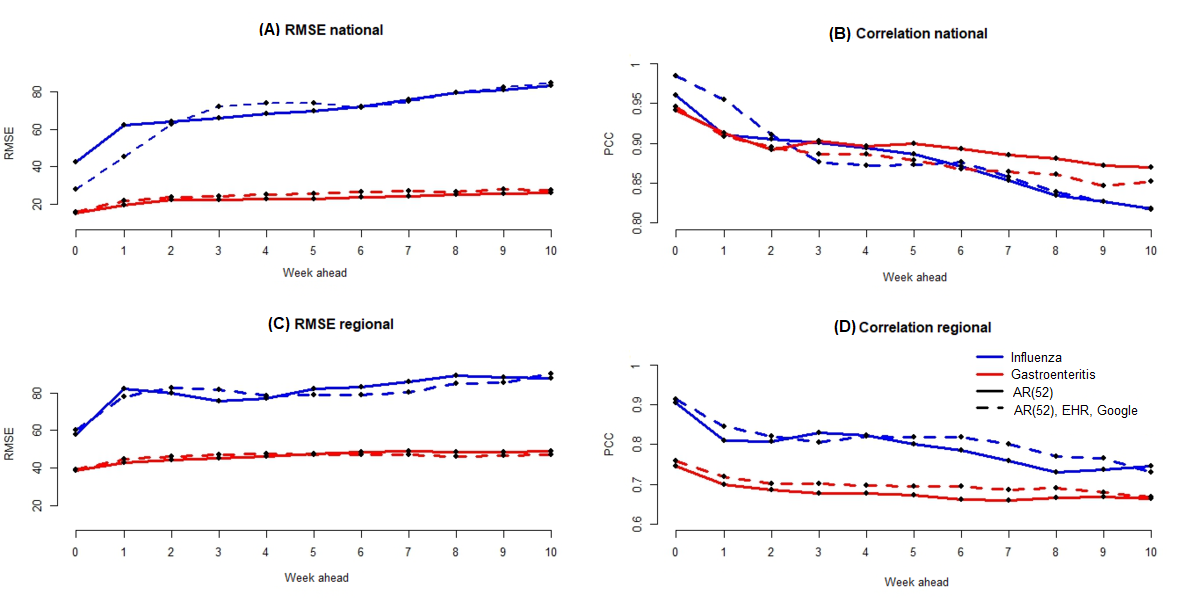
(A) Error values obtained at the national level for the flu and gastroenteritis for forecasts up to 10 weeks with the RF model. The solid line corresponds to the results obtained with the random forest (RF) model using only historical data. The dotted line corresponds to the results obtained with the RF model using historical data and both Google and EHR data. The red color is the results for gastroenteritis disease, whereas the blue color is the results for the flu. This style line and color code are used for the 4 panels of this figure. (B) Correlation values obtained at the national level. (C) Error values obtained at the regional level. (D) Correlation values obtained at the regional level. EHR: electronic health record.

Figure 9 and Table S3 in Multimedia Appendix 1 show, for the nonlinear approach, errors and correlation for AG at the national and regional levels, for forecasts up to 10 weeks. At the national level, in terms of errors, the lowest values are obtained by using only historical data—AR(52). In terms of correlation, for long-term forecasts, the highest values are obtained by using only historical data. At the regional level, in terms of errors, for forecast up to 4 weeks, the lowest values are obtained by using only historical data. However, for long-term forecasts, the most accurate results are obtained by using historical data and both data sources, Google and EHR.

Figure 9 and Table S4 in Multimedia Appendix 1 show, for the nonlinear approach, errors and correlation for influenza at the national and regional levels, for forecasts up to 10 weeks. At the national level, in terms of errors and correlation, the most accurate values for forecasts up to 2 weeks are obtained by using historical data and both Google and EHR data. For forecasts from up to 3 weeks to up to 5 weeks, most accurate estimates are obtained by using only historical data. For long-term forecasts, results are similar for both models, the one using only historical data and the one using historical data and Google and EHR data. At the regional level, for forecasts up to 4 weeks, in terms of errors, the lowest values are obtained, in most cases, by using only historical data. For long-term forecasts, the most accurate estimates are obtained with the model using historical data and both Google and EHR data. In terms of correlation, in most cases, the highest values are obtained by using historical data and both Google and EHR data.

## Discussion

### Principal Findings

We adjusted a methodology developed for influenza, to accurately track AG activity. Our method is able to provide forecasts up to 10 weeks for national and regional levels and for emergency and hospitalization stays (Multimedia Appendix 1). To the best of our knowledge, this is a disease and a spatial resolution (French regions and hospitals) for which no forecasting approaches have been explored previously.

In this study, we show that external data sources, EHR and Google, contribute to improving AG surveillance, in particular for long-term forecasts, with more important contribution from historical data. Specifically, when we use the linear approach (Elastic Net), in terms of errors at the national level, the lowest values are obtained by using historical data and both Google and EHR data. These results are consistent for forecasts from up to 1 week to up to 10 weeks (Table S1 in Multimedia Appendix 1). At the regional level, the model using only historical data is the model producing the lowest errors for short-term forecasts (Table S1 in Multimedia Appendix 1). However, for long-term forecasts, the inclusion of external data sources (Google and EHR) improves the estimates. We conducted a Diebold Mariano test [39] to assess if the forecasts are statistically different when using only historical data or the combination of historical data, Google data, and EHR data (Table S5 in Multimedia Appendix 1). We can see that at the national level, the estimates are statistically more accurate when using historical data and both Google and EHR data for 3-week and long-term forecasts. At the regional level, the use of external data sources produces estimates that are statistically more accurate for 7-week and long-term forecasts.

As we used a method developed for influenza outbreaks, we compared the results obtained for AG with those obtained for influenza. At the national and regional levels, with the linear approach, for both short-term and long-term forecasts, the most accurate estimates are obtained with the model using historical data and external data sources (Google and EHR data). An understanding of these results can emerge from the time series analysis (Figure 7). We show that the seasonality is more important for AG epidemics than for influenza, resulting in historical data capable of providing more information for AG than for influenza. Nonetheless, for long-term forecasts, historical data are not sufficient and external data sources can be used to supplement them. Thus, it is important to integrate external data to improve long-term estimates.

In addition to the linear approach, we conducted the same analysis with a nonlinear approach (RF). At the national level, the results differ slightly from those obtained using the linear approach. In terms of error and correlation, the model using only historical data provides more accurate estimates than the model using historical data, Google data, and EHR data. These results are consistent for real-time estimates and long-term forecasts (Table S3 in Multimedia Appendix 1). At the regional level, regarding the linear approach, in terms of error for short-term forecasts, the model using only historical data allows to produce the most accurate estimates. For long-term forecasts, the model including external data sources, Google and EHR, decreases the error. In terms of correlation, for both short-term and long-term forecasts, the model producing the highest values is the model using historical data, Google data, and EHR data. In all cases, the nonlinear approach allows us to obtain high values in terms of correlation and low values in terms of error when compared with those obtained using the linear approach. However, as seen in Figures 5 and 6, the nonlinear approach tends to smooth the estimates compared with those obtained using the linear approach. This can result in decrease in error and increase in correlation.

The fact that we could only access EHR data from Rennes University Hospital, and thus from the Brittany region, prevented us from being able to quantify the added value of nation-wide EHR information. This should be evaluated in future studies by integrating EHR data from different hospitals from all the French regions. However, it is interesting that data from a hospital in Rennes can improve AG forecasting at the national level, even if, as we described previously, EHR data seem more important for the regional level.

Data retrieved from Google Correlate are normalized by Google in a (frequently) distinct sample and over different time periods depending on the data request. This prenormalization can affect our results, but as shown in the study by Arena et al [15], the process of dynamic training minimizes the impact of this instability.

It would be interesting to test other approaches that gave good results for influenza, for example, an ensemble method that combines the power of the linear and the nonlinear approaches [14] or other machine learning methods such as Support Vector Machine or neural networks. We tested a long short-term memory model to forecast gastroenteritis up to 10 weeks. We obtained root mean squared error=2.96 for real-time forecasting. We believe that these results are really promising and could be further studied in the future by developing a neural network combining long short-term memory for historical data and another neural network for external data sources such as Google data or EHR data. In addition, other methods could be tested to obtain more information from external data sources as transformations of the input variables. Variable transformations could be tested on external data sources to check whether we could get more information. Finally, it could be meaningful to first remove the multicollinearity of our predictive variables with traditional methods such as the Variance Inflation Factor and then select the most important variables with a stepwise regression to run a linear regression on the remaining variables.

### Conclusions

We show that hospital data and internet search data significantly contribute to predict AG outbreaks, in particular for long-term forecasts. The use of these external data sources in combination with historical data could supplement traditional surveillance systems. The methods we developed could help to reduce the impact of the AG peak, particularly in hospitals, by making it possible to anticipate increased activity by up to 10 weeks.

We acknowledge that there is still scope for improvement. Future studies could explore the incorporation of more information from external data sources as a way to yield more robust results.

## Appendix

Multimedia Appendix 1 Heat maps obtained at both, national and regional levels, for the linear approach at 1-week, 2-week and 3-week forecasts. We also added the correlation and errors obtained up to 10-week forecast, for the linear and nonlinear approaches for both, influenza and gastroenteritis diseases.

## Abbreviations

ACF: autocorrelation function
AG: acute gastroenteritis
AR(52): autoregressive model of order 52
CDW: clinical data warehouse
eHOP: entrepôt de données de l’HÔPital
EHR: electronic health record
PACF: partial autocorrelation function
RF: random forest
