# Supplementary material for: Gastroenteritis Forecasting Assessing the Use of Web and Electronic Health Record Data With a Linear and a Nonlinear Approach: Comparison Study
Source: JMIR Public Health Surveill. 2023 Jan 31;9:e34982. doi: 10.2196/34982 (PMC9929730; doi:10.2196/34982)
Supplement: Multimedia Appendix 1 [file publichealth_v9i1e34982_app1.docx]

Multimedia Appendix 1


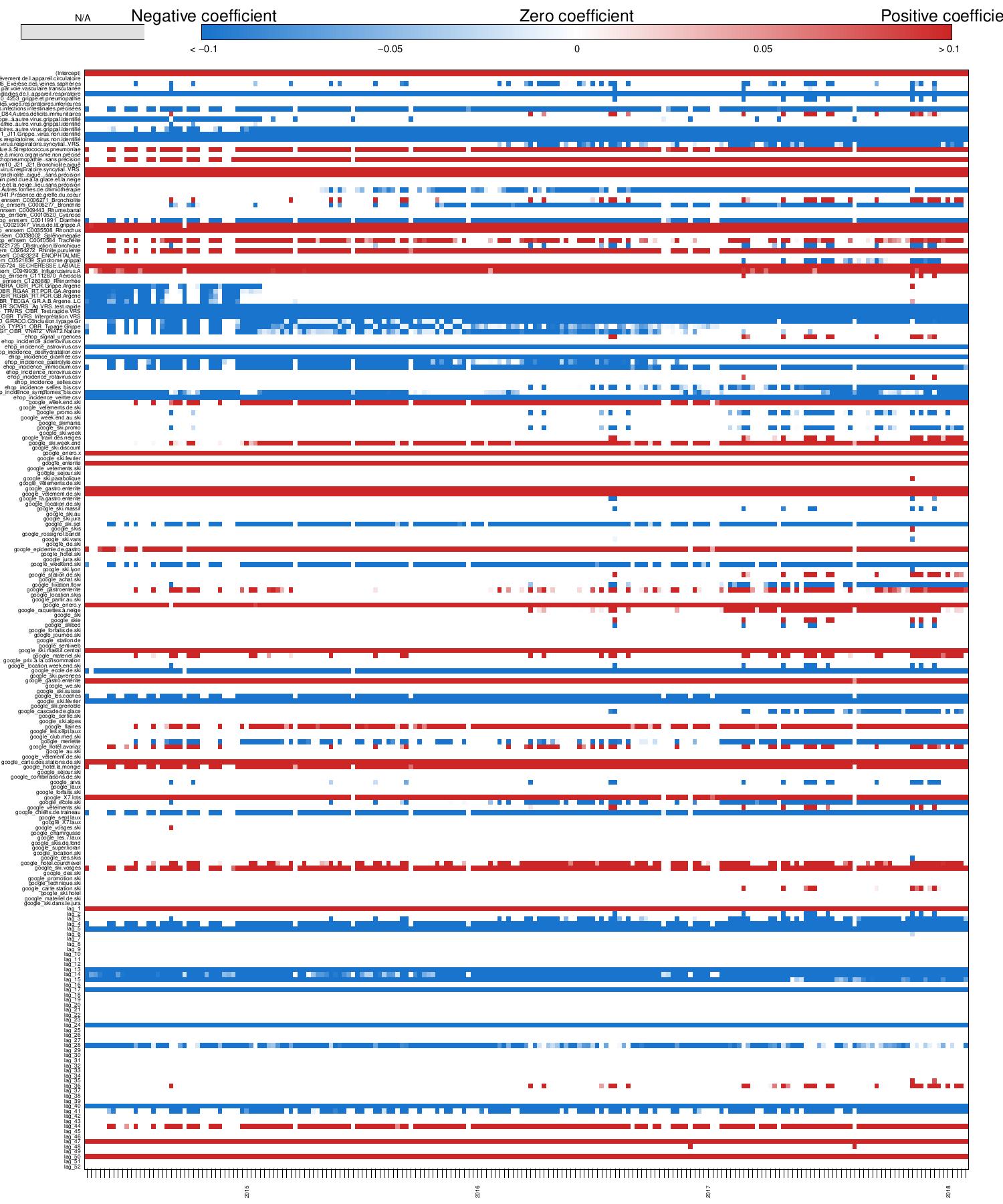


Figure S1. National level. Heatmap of the coefficients for 1-week forecast. Each line of the heatmap corresponds to one predictive variable used in the model and each point of the line corresponds to one week predicted. The first block of variables corresponds to EHR data, the second one corresponds to Google data and the third one to historical data. In blue, a negative coefficient is associated to the variable whereas in red, it is a positive coefficient. The white color means that the predictive variable is not selected by the model and do not participate for forecasting the corresponding week.


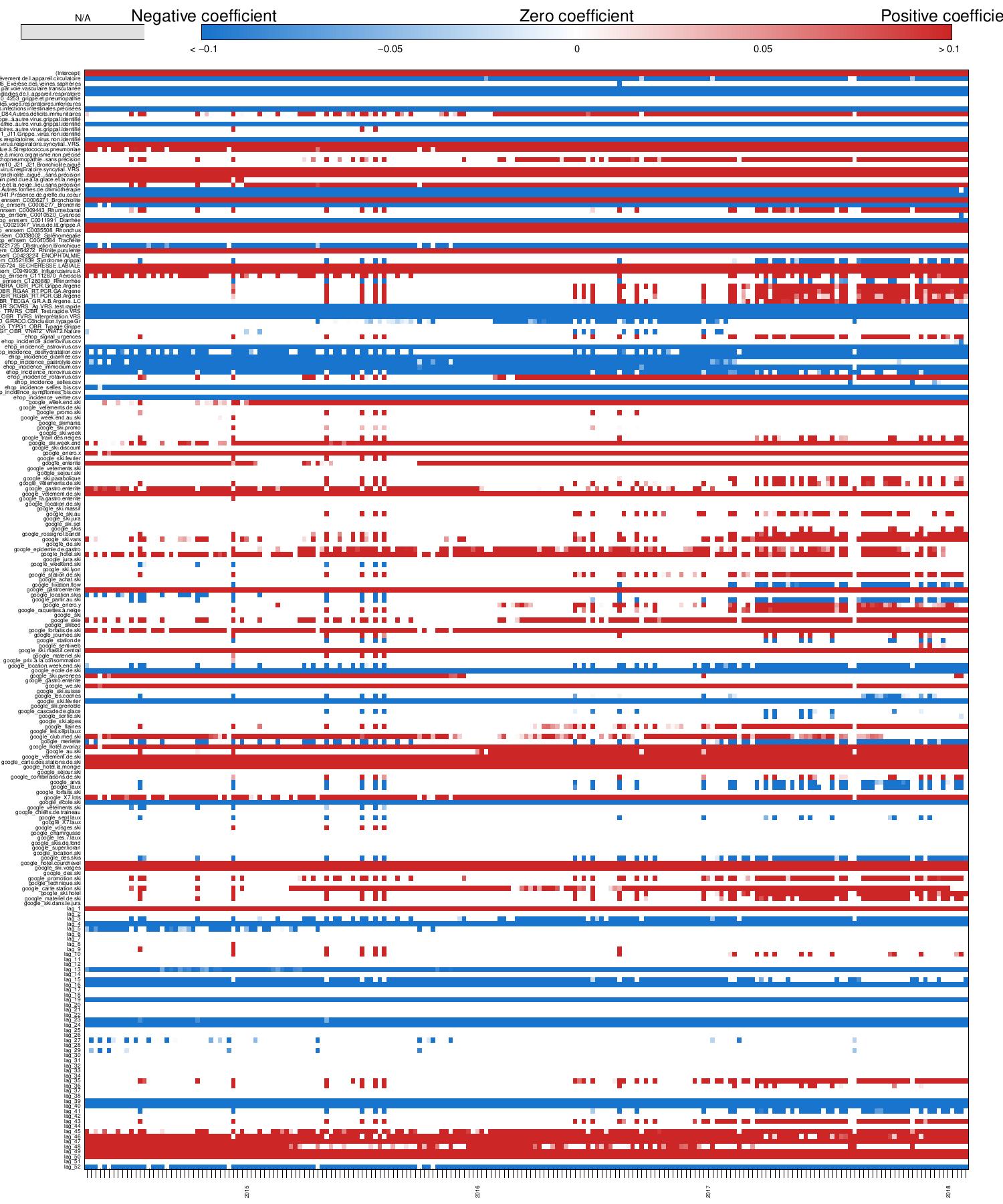


Figure S2. National level. Heatmap of the coefficients for 2-week forecast. Each line of the heatmap corresponds to one predictive variable used in the model and each point of the line corresponds to one week predicted. The first block of variables corresponds to EHR data, the second one corresponds to Google data and the third one to historical data. In blue, a negative coefficient is associated to the variable whereas in red, it is a positive coefficient. The white color means that the predictive variable is not selected by the model and do not participate for forecasting the corresponding week.


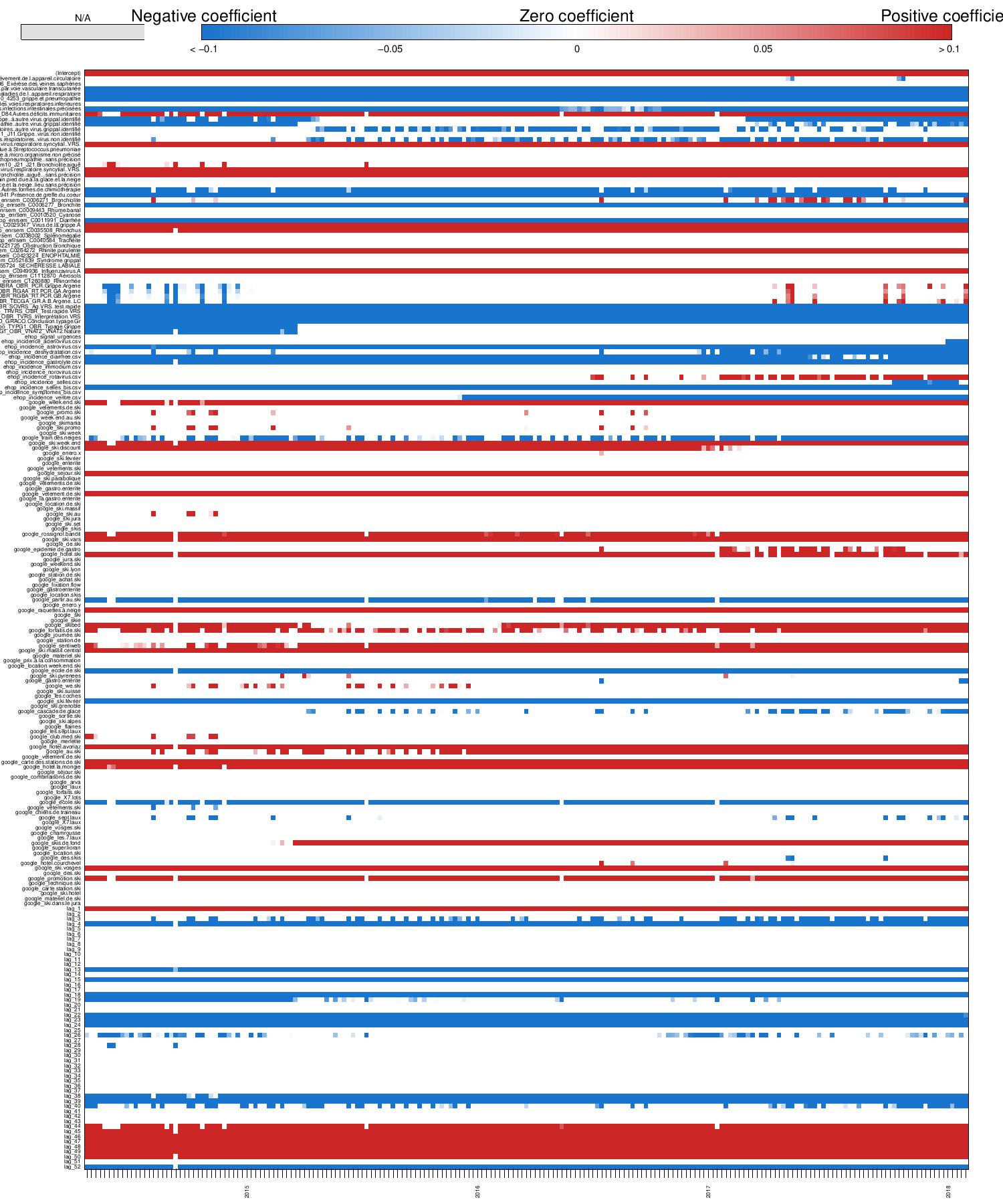


Figure S3. National level. Heatmap of the coefficients for 3-week forecast. Each line of the heatmap corresponds to one predictive variable used in the model and each point of the line corresponds to one week predicted. The first block of variables corresponds to EHR data, the second one corresponds to Google data and the third one to historical data. In blue, a negative coefficient is associated to the variable whereas in red, it is a positive coefficient. The white color means that the predictive variable is not selected by the model and do not participate for forecasting the corresponding week.


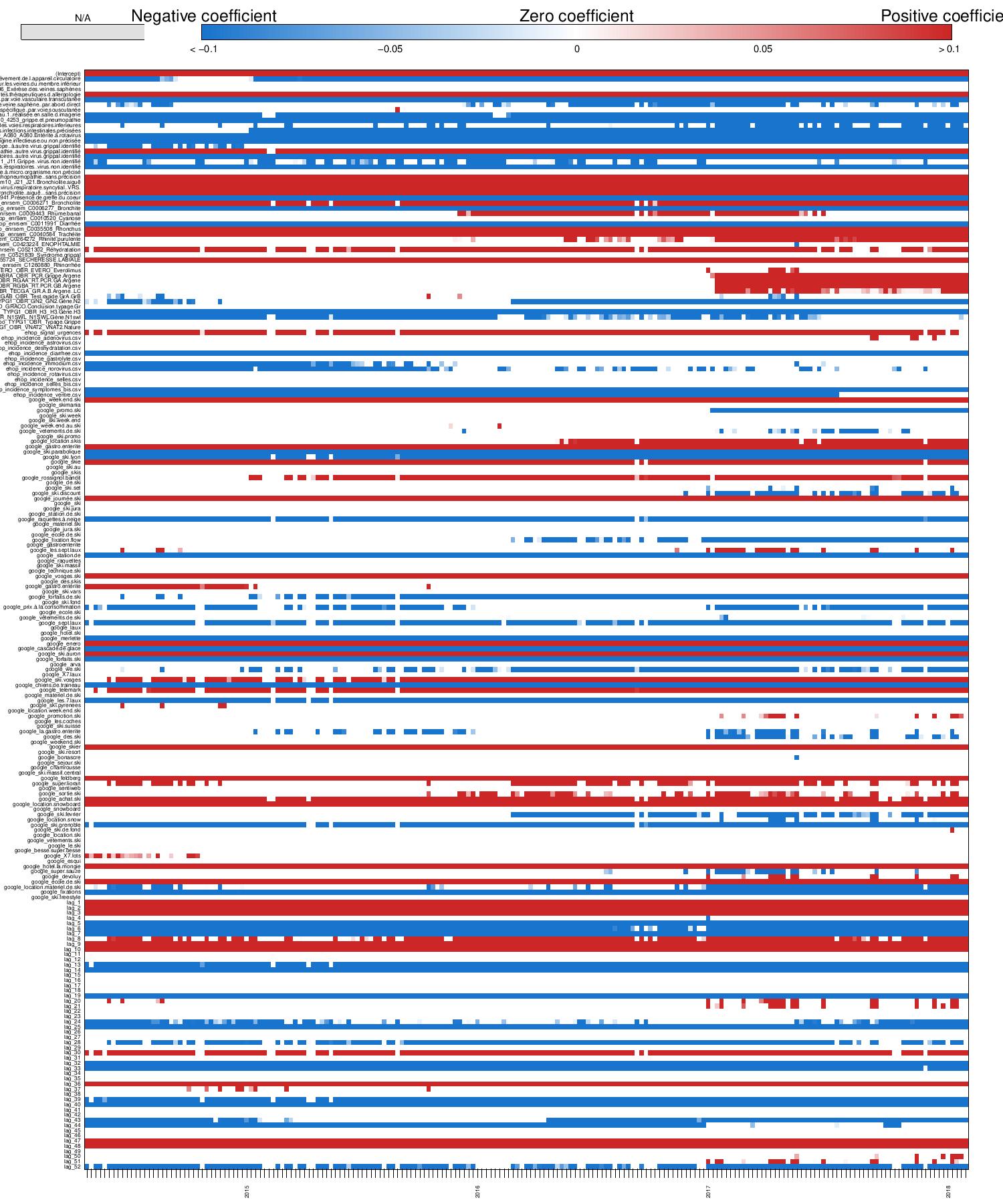


Figure S4. Regional level. Heatmap of the coefficients for 1-week forecast. Each line of the heatmap corresponds to one predictive variable used in the model and each point of the line corresponds to one week predicted. The first block of variables corresponds to EHR data, the second one corresponds to Google data and the third one to historical data. In blue, a negative coefficient is associated to the variable whereas in red, it is a positive coefficient. The white color means that the predictive variable is not selected by the model and do not participate for forecasting the corresponding week.


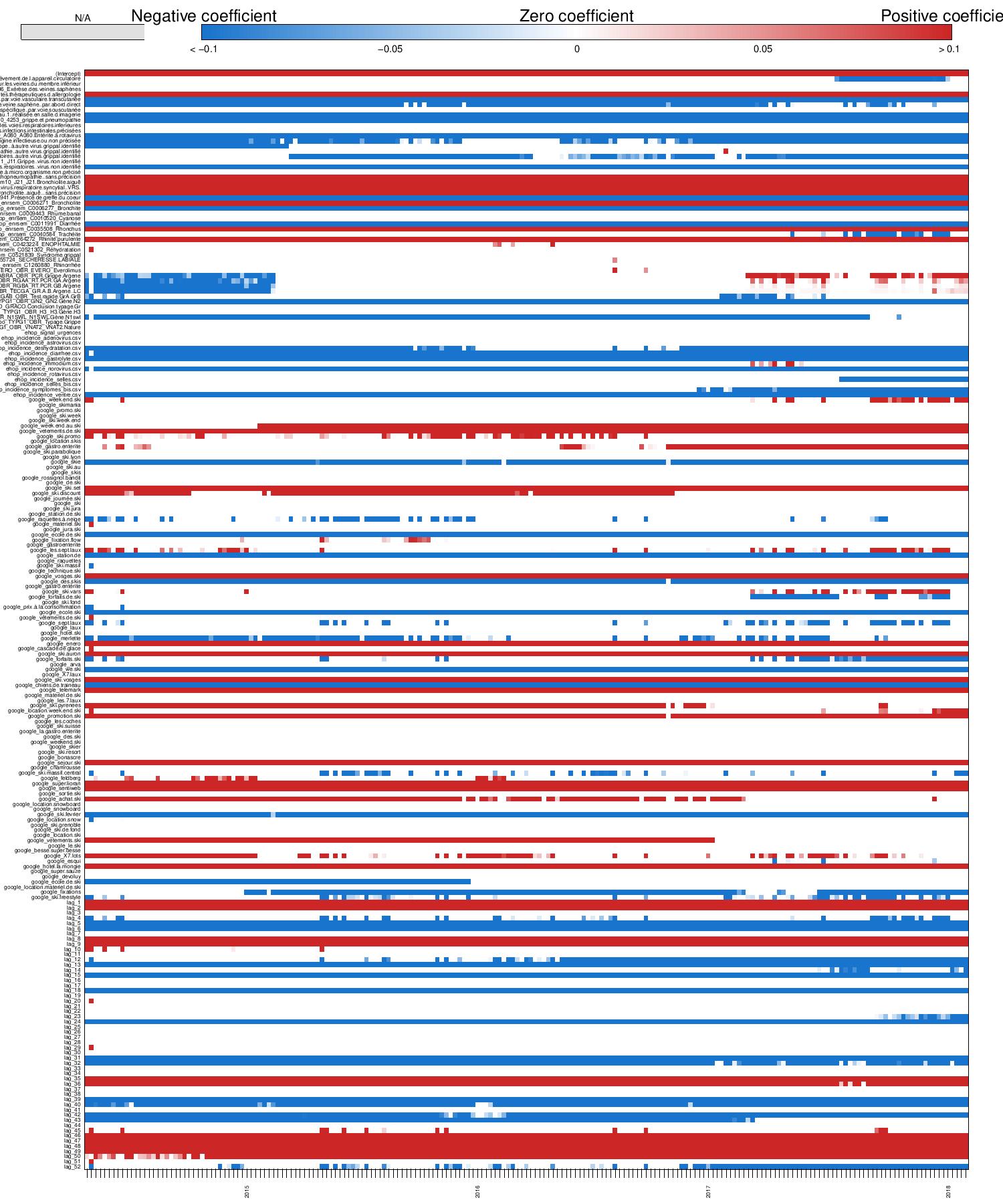


Figure S5. Regional level. Heatmap of the coefficients for 2-week forecast. Each line of the heatmap corresponds to one predictive variable used in the model and each point of the line corresponds to one week predicted. The first block of variables corresponds to EHR data, the second one corresponds to Google data and the third one to historical data. In blue, a negative coefficient is associated to the variable whereas in red, it is a positive coefficient. The white color means that the predictive variable is not selected by the model and do not participate for forecasting the corresponding week.


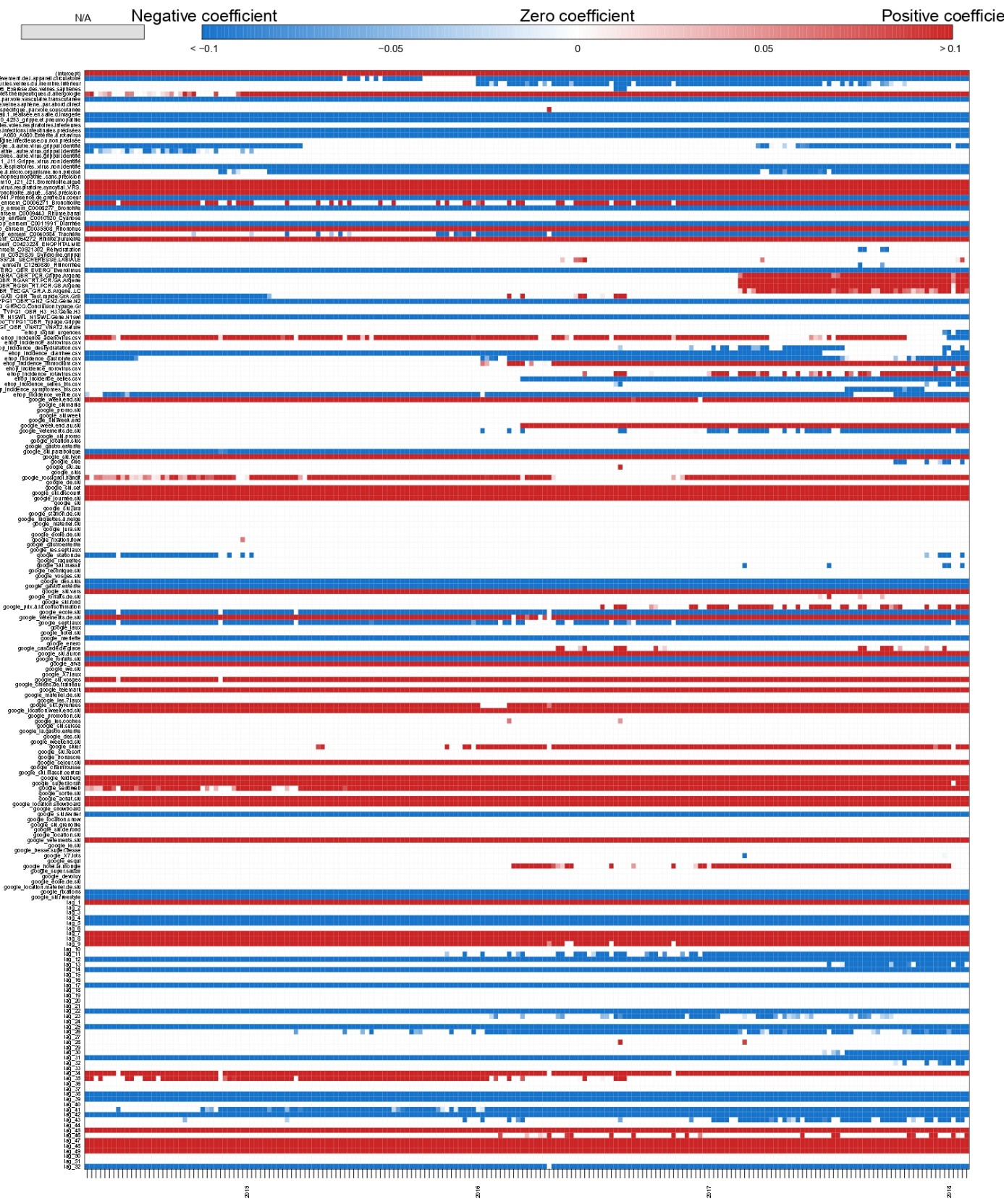


Figure S6. Regional level. Heatmap of the coefficients for 3-week forecast. Each line of the heatmap corresponds to one predictive variable used in the model and each point of the line corresponds to one week predicted. The first block of variables corresponds to EHR data, the second one corresponds to Google data and the third one to historical data. In blue, a negative coefficient is associated to the variable whereas in red, it is a positive coefficient. The white color means that the predictive variable is not selected by the model and do not participate for forecasting the corresponding week.

Table S1. National and regional levels Errors and correlation obtained for AG for forecasts up to 10 weeks with the linear approach

| Forecast | Real-time | 1-week | 2-week | 3-week | 4-week | 5-week | 6-week | 7-week | 8-week | 9-week | 10-week |
| --- | --- | --- | --- | --- | --- | --- | --- | --- | --- | --- | --- |
| **National level** | | | | | | | | | | | |
| **RMSE**  AR(52) | **16.16** | 22.69 | 26.95 | 30.69 | 34.07 | 36.35 | 38.29 | 39.82 | 41.13 | 42.33 | 42.90 |
| **RMSE**  AR(52)  Google EHR | 21.26 | **20.94** | **24.16** | **25.33** | **29.80** | **31.91** | **32.61** | **32.12** | **32.07** | **34.56** | **32.47** |
| **PCC**  AR(52) | **0.946** | **0.910** | **0.898** | **0.884** | **0.873** | **0.869** | **0.863** | **0.856** | **0.851** | **0.848** | **0.851** |
| **PCC**  AR(52)  Google EHR | 0.936 | 0.903 | 0.856 | 0.845 | 0.802 | 0.791 | 0.799 | 0.808 | 0.805 | 0.767 | 0.784 |
| **Regional level** | | | | | | | | | | | |
| **RMSE**  AR(52) | **40.75** | **44.18** | 47.65 | **49.12** | 52.37 | 54.63 | 57.44 | 60.91 | 61.56 | 62.53 | 63.93 |
| **RMSE**  AR(52)  Google EHR | 42.12 | 45.24 | **47.37** | 52.19 | **51.96** | **51.31** | **56.18** | **53.03** | **54.08** | **55.66** | **54.75** |
| **PCC**  AR(52) | **0.725** | **0.705** | **0.670** | **0.681** | **0.670** | **0.665** | **0.657** | **0.631** | **0.641** | **0.634** | **0.634** |
| **PCC**  AR(52)  Google EHR | 0.724 | 0.689 | 0.646 | 0.620 | 0.615 | 0.633 | 0.580 | 0.616 | 0.612 | 0.577 | 0.563 |

Table S2. National and regional levels Errors and correlation obtained for the flu for forecasts up to 10 weeks with the linear approach

| Forecast | Real-time | 1-week | 2-week | 3-week | 4-week | 5-week | 6-week | 7-week | 8-week | 9-week | 10-week |
| --- | --- | --- | --- | --- | --- | --- | --- | --- | --- | --- | --- |
| **National level** | | | | | | | | | | | |
| **RMSE**  AR(52) | 31.60 | 64.51 | 90.60 | 108.19 | 117.13 | 120.58 | 121.23 | 122.08 | 122.73 | 122.76 | 122.84 |
| **RMSE**  AR(52)  Google EHR | **30.18** | **63.98** | **86.80** | **96.81** | **99.77** | **100.43** | **99.49** | **109.55** | **111.54** | **117.63** | **121.03** |
| **PCC**  AR(52) | 0.979 | 0.906 | 0.800 | 0.698 | 0.633 | 0.601 | 0.579 | 0.555 | 0.544 | 0.544 | 0.545 |
| **PCC**  AR(52)  Google EHR | **0.980** | **0.910** | **0.820** | **0.766** | **0.744** | **0.735** | **0.731** | **0.666** | **0.646** | **0.610** | **0.609** |
| **Regional level** | | | | | | | | | | | |
| **RMSE**  AR(52) | 54.85 | 81.70 | 96.76 | 105.85 | 109.81 | 111.83 | 110.86 | 110.29 | 110.69 | 110.74 | 110.56 |
| **RMSE**  AR(52)  Google EHR | **53.42** | **80.97** | **87.40** | **95.06** | **97.94** | **103.98** | **105.65** | **94.80** | **92.63** | **99.55** | **106.30** |
| **PCC**  AR(52) | 0.916 | 0.803 | 0.702 | 0.626 | 0.587 | 0.563 | 0.551 | 0.537 | 0.524 | 0.524 | 0.528 |
| **PCC**  AR(52)  Google EHR | **0.920** | **0.810** | **0.766** | **0.708** | **0.684** | **0.653** | **0.631** | **0.697** | **0.706** | **0.667** | **0.635** |

Table S3. National and regional levels Errors and correlation obtained for AG for forecasts up to 10 weeks with the non linear approach

| Forecast | Real-time | 1-week | 2-week | 3-week | 4-week | 5-week | 6-week | 7-week | 8-week | 9-week | 10-week |
| --- | --- | --- | --- | --- | --- | --- | --- | --- | --- | --- | --- |
| **National level** | | | | | | | | | | | |
| **RMSE**  AR(52) | **15.47** | **19.71** | **22.19** | **22.30** | **23.07** | **22.99** | **23.84** | **24.41** | **24.99** | **25.87** | **26.09** |
| **RMSE**  AR(52)  Google EHR | 15.72 | 21.76 | 23.87 | 24.41 | 25.03 | 25.55 | 26.59 | 26.94 | 26.59 | 27.86 | 27.33 |
| **PCC**  AR(52) | 0.942 | **0.913** | 0.892 | **0.903** | **0.896** | **0.900** | **0.893** | **0.885** | **0.881** | **0.872** | **0.870** |
| **PCC**  AR(52)  Google EHR | **0.946** | 0.909 | **0.895** | 0.886 | 0.886 | 0.879 | 0.868 | 0.864 | 0.861 | 0.847 | 0.852 |
| **Regional level** | | | | | | | | | | | |
| **RMSE**  AR(52) | **38.47** | **42.68** | **44.11** | **45.05** | **46.08** | 47.34 | 48.60 | 48.76 | 48.49 | 48.32 | 48.76 |
| **RMSE**  AR(52)  Google EHR | 38.88 | 44.63 | 46.25 | 47.17 | 47.34 | **47.14** | **46.85** | **47.08** | **46.01** | **46.43** | **47.05** |
| **PCC**  AR(52) | 0.745 | 0.699 | 0.685 | 0.677 | 0.678 | 0.672 | 0.661 | 0.660 | 0.665 | 0.668 | 0.664 |
| **PCC**  AR(52)  Google EHR | **0.759** | **0.718** | **0.702** | **0.701** | **0.696** | **0.694** | **0.695** | **0.686** | **0.690** | **0.679** | **0.668** |

Table S4. National and regional levels Errors and correlation obtained for the flu for forecasts up to 10 weeks with the non linear approach

| Forecast | Real-time | 1-week | 2-week | 3-week | 4-week | 5-week | 6-week | 7-week | 8-week | 9-week | 10-week |
| --- | --- | --- | --- | --- | --- | --- | --- | --- | --- | --- | --- |
| **National level** | | | | | | | | | | | |
| **RMSE**  AR(52) | 42.50 | 62.07 | 64.00 | **65.82** | **68.21** | **69.51** | 72.20 | 75.70 | 79.74 | **81.18** | **83.37** |
| **RMSE**  AR(52)  Google EHR | **28.23** | **45.17** | **62.80** | 72.05 | 74.01 | 73.91 | **71.55** | **75.08** | **79.42** | 82.18 | 84.71 |
| **PCC**  AR(52) | 0.960 | 0.911 | 0.905 | **0.901** | **0.894** | **0.887** | 0.871 | 0.853 | 0.835 | **0.827** | **0.818** |
| **PCC**  AR(52)  Google EHR | **0.985** | **0.955** | **0.911** | 0.877 | 0.872 | 0.873 | **0.877** | **0.858** | **0.839** | **0.827** | **0.817** |
| **Regional level** | | | | | | | | | | | |
| **RMSE**  AR(52) | **57.89** | 82.17 | **79.93** | **75.80** | **77.20** | 82.36 | 83.29 | 86.11 | 89.14 | 88.27 | **87.59** |
| **RMSE**  AR(52)  Google EHR | 60.18 | **77.97** | 82.57 | 81.76 | 78.38 | **78.90** | **78.67** | **80.50** | **84.78** | **85.23** | 89.94 |
| **PCC**  AR(52) | 0.904 | 0.809 | 0.808 | **0.829** | **0.823** | 0.800 | 0.786 | 0.758 | 0.729 | 0.737 | **0.745** |
| **PCC**  AR(52)  Google EHR | **0.912** | **0.844** | **0.821** | 0.805 | 0.820 | **0.819** | **0.818** | **0.801** | **0.770** | **0.766** | 0.731 |

Table S5. Results of the Diebold Mariano test to assess if the forecasts up to 10 weeks are statistically different by using only historical data or the combination of historical data, Google and EHR data. In red, the p-values for which the estimates are statistically different.

| p-value | Real-time | 1-wk | 2-wk | 3-wk | 4-wk | 5-wk | 6-wk | 7-wk | 8-wk | 9-wk | 10-wk |
| --- | --- | --- | --- | --- | --- | --- | --- | --- | --- | --- | --- |
| National | 0.15 | 0.19 | 0.05 | 0.0004 | 0.009 | 0.01 | 0.001 | 2*10^-5^ | 9*10^-7^ | 0.0001 | 2*10^-8^ |
| Regional | 0.20 | 0.25 | 0.91 | 0.28 | 0.89 | 0.24 | 0.6 | 0.003 | 0.004 | 0.007 | 0.003 |

## Prediction of emergency and hospital activity

## *Emergency level*

We calculated from eHOP data, the number of patients per week coming to the emergency department due to AG. In April 2018, we retrieved this signal from January 2008 to March 2018.

## *Hospital level*

We calculated from eHOP data, the number of patients per week who were hospitalized after their passage to the emergency department for AG. We retrieved this signal from January 2008 to March 2018 in April 2018.

Because hospital data are available in near real time, we applied the linear approach to forecast from up to one-week to
up to three-week.

Table S6. PCC and RMSE values obtained for the entire prediction period (May 2014 to March 2018) for Emergency and Hospital levels.

|  | Real-time | | 1-week forecast | | 2-week forecast | | 3-week forecast | |
| --- | --- | --- | --- | --- | --- | --- | --- | --- |
|  | PCC | RMSE | PCC | RMSE | PCC | RMSE | PCC | RMSE |
| **Emergency** |  | | | | | | | |
| AR(52) |  |  | 0.774 | 1,24 ∗ 10^−3^ | 0.713 | 1,37 ∗ 10^−3^ | 0.693 | 1,40 ∗ 10^−3^ |
| Google |  |  | 0.741 | 1,38 ∗ 10^−3^ | **0.769** | 1,28 ∗ 10^−3^ | 0.761 | 1,33 ∗ 10^−3^ |
| EHR |  |  | 0.674 | 1,55 ∗ 10^−3^ | 0.644 | 1,67 ∗ 10^−3^ | 0.645 | 1,52 ∗ 10^−3^ |
| AR(52)+Google |  |  | 0.783 | 1,21 ∗ 10^−3^ | 0.758 | 1,27 ∗ 10^−3^ | 0.759 | 1,28 ∗ 10^−3^ |
| AR(52)+EHR |  |  | 0.776 | 1,22 ∗ 10^−3^ | 0.724 | 1,34 ∗ 10^−3^ | 0.717 | 1,36 ∗ 10^−3^ |
| Google+EHR |  |  | 0.732 | 1,35 ∗ 10^−3^ | 0.742 | 1,31 ∗ 10^−3^ | 0.757 | 1,28 ∗ 10^−3^ |
| AR(52)+Google+EHR |  |  | **0.792** | **1,19 ∗ 10^−3^** | 0.765 | **1,25 ∗ 10^−3^** | **0.767** | **1,25 ∗ 10^−3^** |
| **Hospital stay** |  | | | | | | | |
| AR(52) |  |  | 0.669 | 3,30 ∗ 10^−4^ | 0.662 | 3,34 ∗ 10^−4^ | 0.661 | 3,39 ∗ 10^−4^ |
| Google |  |  | **0.740** | **3,00 ∗ 10^−4^** | **0.723** | **3,13 ∗ 10^−4^** | 0.733 | 3,09 ∗ 10^−4^ |
| EHR |  |  | 0.622 | 3,51 ∗ 10^−4^ | 0.598 | 3,86 ∗ 10^−4^ | 0.613 | 3,69 ∗ 10^−4^ |
| AR(52)+Google |  |  | 0.707 | 3,14 ∗ 10^−4^ | 0.710 | **3,14 ∗ 10^−4^** | 0.723 | 3,09 ∗ 10^−4^ |
| AR(52)+EHR |  |  | 0.688 | 3,23 ∗ 10^−4^ | 0.679 | 3,29 ∗ 10^−4^ | 0.626 | 3,49 ∗ 10^−4^ |
| Google+EHR |  |  | 0.738 | 3,02 ∗ 10^−4^ | **0.722** | **3,12 ∗ 10^−4^** | **0.739** | **3,04 ∗ 10^−4^** |
| AR(52)+Google+EHR |  |  | 0.725 | 3,07 ∗ 10^−4^ | 0.714 | **3***,***14** ∗ **10**−**4** | 0.734 | **3***,***06** ∗ **10**−**4** |


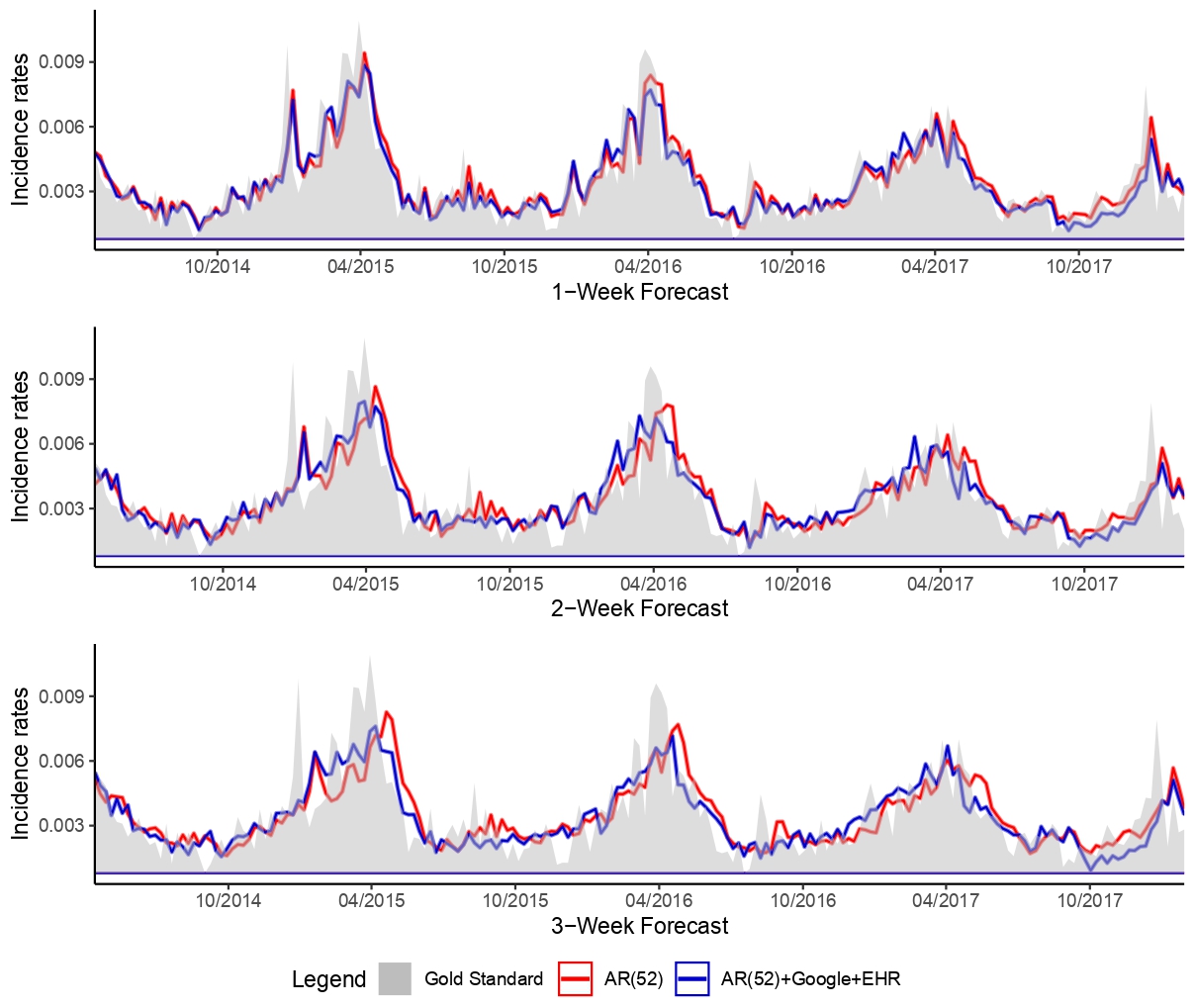


Figure S7. Emergency department level. Predictions up to three weeks obtained at the emergency department level. Gold standard, AG incidence rates from the emergency department of Rennes' hospital (Brittany, France).


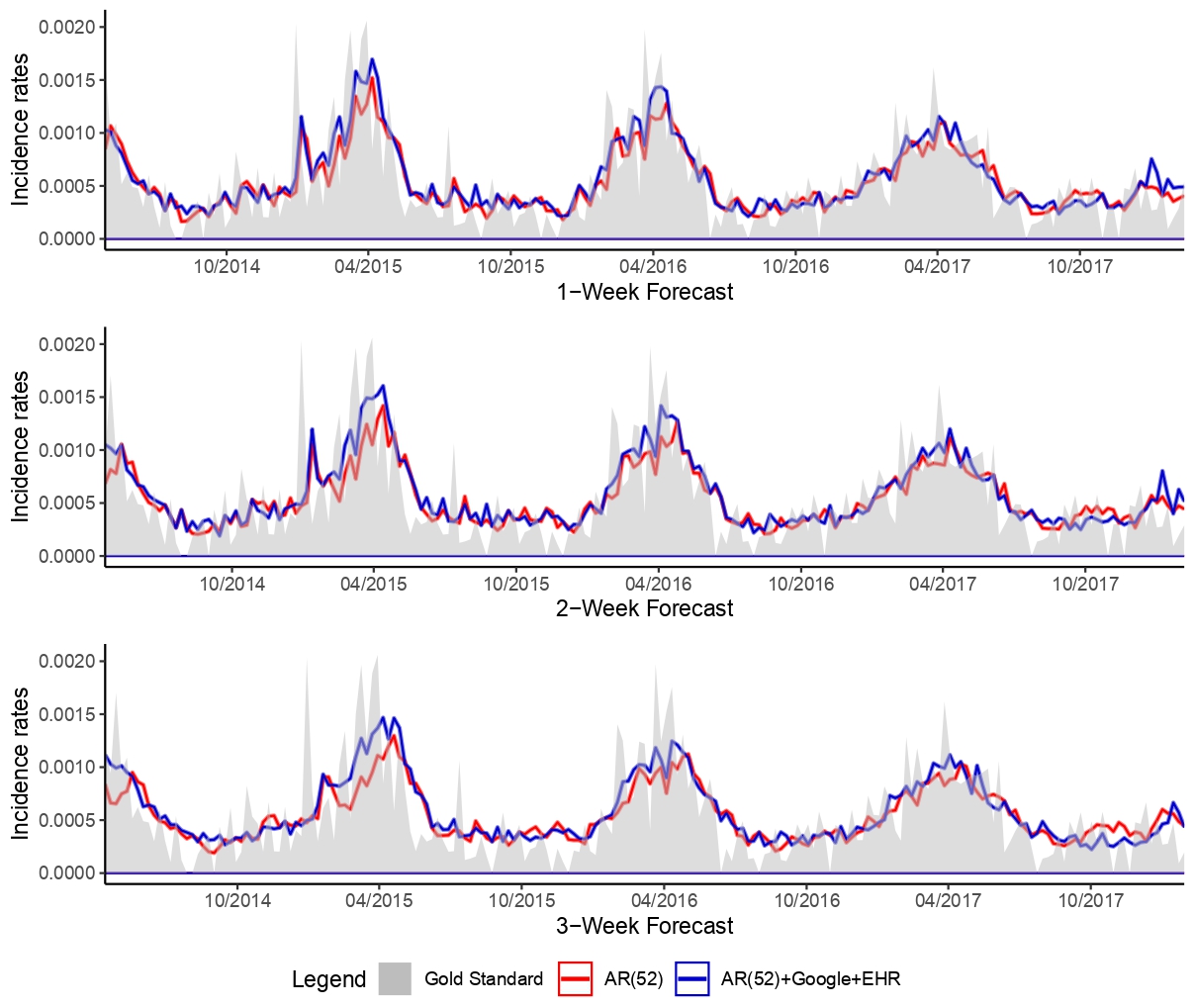


Figure S8. Hospital level. Predictions up to three weeks obtained at the hospital level. Gold standard, AG incidence rates from hospital stays of Rennes' hospital (Brittany, France).
